# A Novel MFS-MDR Transporter, MdrP, Employs D223 as a Key Determinant in the Na^+^ Translocation Coupled to Norfloxacin Efflux

**DOI:** 10.3389/fmicb.2020.00955

**Published:** 2020-05-29

**Authors:** Rui Zhang, Heba Abdel-Motaal, Qiao Zou, Sijia Guo, Xiutao Zheng, Yuting Wang, Zhenglai Zhang, Lin Meng, Tong Xu, Juquan Jiang

**Affiliations:** Department of Microbiology and Biotechnology, College of Biological Sciences, Northeast Agricultural University, Harbin, China

**Keywords:** MFS transporter, multidrug resistance, H^+^ coupling, Na^+^ coupling, antiporter

## Abstract

Multidrug resistance (MDR) transporters of the major facilitator superfamily (MFS) were previously believed to drive the extrusion of multiple antimicrobial drugs through the coupling to proton translocation. Here, we present the identification of the first Na^+^-coupled MFS-MDR transporter, MdrP, which also can achieve H^+^-coupled drug efflux independently of Na^+^. Importantly, we propose that MdrP can extrude norfloxacin in a mode of drug/Na^+^ antiport, which has not yet been reported in any MFS member. On this basis, we further provide the insights into a novel Na^+^ and H^+^ coupling mechanism of MFS-MDR transporters, even for all secondary transporters. The most important finding lies in that D223 should mainly act as a key determinant in the Na^+^ translocation coupled to norfloxacin efflux. Furthermore, our results partially modify the knowledge of the conformational stability-related residues in the motif A of MFS transporters and imply the importance of a new positively charged residue, R361, for the stabilization of outward-facing conformation of MFS transporters. These novel findings positively contribute to the knowledge of MFS-MDR transporters, especially about Na^+^ and H^+^ coupling mechanism. This study is based mainly on measurements in intact cells or everted membranes, and a biochemical assay with a reconstituted MdrP protein should be necessary to come to conclusion to be assured.

## Introduction

The extrusion of clinically relevant antimicrobial drugs mediated by multidrug resistance (MDR) transporters is a major mechanism causing multidrug resistance, which seriously and continuingly threatens medical treatment by bacterial infections (Higgins, 2007; Fluman and Bibi, 2009). These MDR transporters are classified into six families/superfamilies including ATP-binding cassette (ABC) superfamily (Lubelski et al., 2007), resistance–nodulation–division (RND) family (Tseng et al., 1999), small multidrug resistance (SMR) family (Bay et al., 2008), multidrug and toxic compound extrusion (MATE) family (Brown et al., 1999; Kuroda and Tsuchiya, 2009), proteobacterial antimicrobial compound efflux (PACE) family (Hassan et al., 2015), and major facilitator superfamily (MFS) (Reddy et al., 2012). Among them, MFS comprises the largest class of MDR transporters, which were previously believed to drive the extrusion of multiple antimicrobial drugs through the coupling to proton translocation in a mode of drug/H^+^ antiport (Fluman and Bibi, 2009). However, only a small number of MFS-MDR transporters have been identified to be H^+^ antiport driven, such as *Lactococcus lactis* LmrP, *Escherichia coli* MdfA and EmrD (Bolhuis et al., 1995; Mine et al., 1998; Wong et al., 2014). MFS-MDR transporters are categorized into four major drug/H^+^ antiporter (DHA1-4) families with 214 representatives in the Transporter Classification Database (TCDB) (Saier et al., 2016). None of the identified MFS-MDR transporters have been found to be Na^+^ antiport driven. Na^+^-coupled symporters are also rarely found in the families within MFS, with the exception of the anion:cation symporter (ACS) family and glycoside–pentoside–hexuronide:cation symporter (GPH) family (Shi, 2013; Ethayathulla et al., 2014).

The majority of MFS transporters shares a common structural fold designated as MFS fold, in which 12 transmembrane helices (TMHs) are equally divided and organized into two discretely folded domains: N domain with TMH1-6 and C domain with TMH7-12 (Yan, 2013a). The N and C domains form a central cavity in the transmembrane core, with the cytoplasmic side closed and the periplasmic side accessible to the solvent (Jiang et al., 2013; Yan, 2013a). MFS transporters are proposed to transport substrates in a rocker-switch-like “alternating access” mechanism by exposing drug-binding sites to the outside or inside of cells, accompanied with the conformational switch between outward- and inward-facing states (Yan, 2013b). Furthermore, MFS transporters contain a highly conserved “motif A” with the signature sequence of GxLaDrxGrkxxl (Griffith et al., 1992; Paulsen et al., 1996). The structural analysis of *E. coli* YajR, a putative proton-coupled MFS transporter, provides a good evidence for the vital role of motif A in the stabilization of outward-facing conformation of MFS transporters (Jiang et al., 2013). This motif is the most significant feature to be differentiated MFS transporters from MATE transporters, in addition to low sequence homology (Griffith et al., 1992; Paulsen et al., 1996). However, MATE transporters share similar structural characteristics with MFS transporters, despite a significantly different topology of helical arrangement (He et al., 2010; Lu et al., 2013a, b; Tanaka et al., 2013). For example, 12 TMHs of *Vibrio cholerae* NorM are arranged into two domains, N and C domains, each of which is composed of six consecutive TMHs. The N and C domains also form a similar central cavity to those of MFS transporters (He et al., 2010). MATE transporters have been increasingly suggested or established to be able to achieve the extrusion of multiple antimicrobial drugs driven by either sodium motive force or/and proton motive force, such as *Bacillus halodurans* DinF (Lu et al., 2013a), NorM from *Vibrio parahaemolyticus* (Morita et al., 2000), *Neissaeria gonorrhoeae* (Lu et al., 2013b), and *V. cholerae* (Jin et al., 2014). The H^+^ or/and Na^+^ coupling mechanisms of the drug extrusion have been proposed in several MATE transporters (He et al., 2010; Lu et al., 2013a, b; Tanaka et al., 2013; Mousa et al., 2016; Nishima et al., 2016; Krah and Zachariae, 2017; Mousa et al., 2017; Krah et al., 2020).

In our recent study (Abdel-Motaal et al., 2018), an uncharacterized MFS transporter from *Planococcus maritimus* has been identified to function as a Na^+^(Li^+^, K^+^)/H^+^ antiporter and also as a multidrug efflux transporter with the resistance against multiple antimicrobial drugs, such as norfloxacin (Abdel-Motaal et al., 2018). In addition, this transporter was suggested to maybe belong to DHA1 family within MFS (Abdel-Motaal et al., 2018). Accumulation of norfloxacin in *E. coli* CM2/pET-mdrP was found to be significantly enhanced by the addition of carbonyl cyanide m-chlorophenylhydrazone (CCCP), a proton gradient uncoupler. However, accumulation of norfloxacin in *E. coli* CM2/pET-mdrP was unable to reach the same maximum level as that of CM2/pET19 after the addition of CCCP (Abdel-Motaal et al., 2018). Considering MdrP being a Na^+^/H^+^ antiporter, we hypothesize that MdrP may bind with Na^+^ and therefore employ it as a coupling ion to drive norfloxacin efflux in a mode of drug/Na^+^ antiport, similarly to those of Na^+^-coupled MATE transporters, *N. gonorrhoeae* NorM (Lu et al., 2013b) and *V. cholerae* NorM (Jin et al., 2014).

To test this hypothesis, we address here the question whether MdrP can couple Na^+^ or solely H^+^ to norfloxacin efflux. As a result, MdrP was identified to function as a Na^+^-coupled MFS-MDR transporter and also be able to achieve H^+^-coupled drug efflux independently of Na^+^. Importantly, we propose that MdrP can extrude norfloxacin in a mode of drug/Na^+^ antiport, which has not yet been reported in any MFS member. On this basis, we further present the functional and structural roles of three acidic residues and two alkaline residues. Notably, D233 could mainly act as a key determinant in the Na^+^ translocation coupled to norfloxacin efflux, whereas D127 and D244 may be involved in H^+^ or/and Na^+^ translocation. Furthermore, R71, D127, and R361 may be responsible for the stabilization of the outward-facing conformation of MdrP. These novel findings positively contribute to the knowledge of MFS-MDR transporters, especially about Na^+^ and H^+^ coupling mechanism.

## Materials and Methods

### Bacterial Strains, Plasmids, and Growth Conditions

The strains and plasmids in this study are listed in Table 1. The transformants of *E. coli* CM2 (Abdel-Motaal et al., 2018) were grown at 37°C in Luria–Bertani (LB) broth containing 1.0% tryptone (Oxoid Ltd., United Kingdom), 0.5% yeast extract (Oxoid Ltd., United Kingdom), 1.0% NaCl, or LBO (LB without the addition of NaCl) broth supplemented with 50 μg/ml ampicillin and 50 μg/ml chloramphenicol. To analyze the effects of NaCl concentrations on norfloxacin resistance, precultures of *E. coli* CM2 transformants were grown in LBO broths to OD_600 nm_ of 1.0, and then 1% of the precultures continued to be grown in LBO broths containing the indicated NaCl concentrations supplemented with or without 0.025 μg/ml norfloxacin. The transformants of *E. coli* KNabc (Nozaki et al., 1996) were grown at 37°C in LBK (LB with NaCl replaced by 89 mM KCl) broths containing 0.2 M NaCl plus 50 μg/ml ampicillin. To analyze effects of norfloxacin concentrations on NaCl tolerance, precultures of *E. coli* KNabc transformants were grown in LBK broths to OD_600 *nm*_ of 1.0, and then, 1% of the precultures continued to be grown in LBK broths containing 0.2 M NaCl supplemented with the indicated norfloxacin concentrations. Cell growth was ended within 24 h for CM2 or 48 h for KNabc, followed by the evaluation of OD_600 *nm*_.

**TABLE 1:** Strains and plasmids used in this study.

Strains and plasmids	Relevant phenotype or genotype	The source
**Strains**		
*Escherichia coli* KNabc	*nhaA*::Km^*R*^, *nhaB*::Em^*R*^, *chaA*::Cm^*R*^	Donated by Dr. Terry A. Krulwich (Nozaki et al., 1996)
*Escherichia coli* CM2	A major multidrug efflux system-deficient *E. coli* DH5α mutant (Δ*acrAB*)	Constructed in our recent study (Abdel-Motaal et al., 2018)
**Plasmids**		
pEASY T3	Cloning vector	Takara Biotechnology (Dalian) Co., Ltd., China
pUC-PM29	pUC18 carrying a 3.2 kb DNA fragment including 5′-end truncated *uvrD* and *mdrP*	Constructed in our recent study (Abdel-Motaal et al., 2018)
pUC-nhaD	pUC18 carrying *nhaD* with its native promoter	Constructed in our previous study (Wang et al., 2017)
pEASY T3-mdrP	pEASY T3 carrying *mdrP* with its native promoter, and designated as MdrP while used with the strain names	This study
pEASY T3-ydhE	pEASY T3 carrying *ydhE* with its native promoter, and designated as YdhE while used with the strain names	This study
pEASY T3-nhaD	pEASY T3 carrying *nhaD* with its native promoter, and designated as NhaD while used with the strain names	This study

### Gene Cloning and Mutagenesis

The full-length *mdrP* gene preceded by its native promoter was subcloned from the original recombinant plasmid pUC-PM29 (Abdel-Motaal et al., 2018) into the cloning vector pEASY T3 (Transgen Biotech, Beijing, China), together with the fusion of a C-terminal 6 × His tag. Site-directed variants were produced by the Fast Mutagenesis System Kit (Transgen Biotech, Beijing, China) with the plasmid pEASY T3-mdrP as the template and the mutation primers listed in Supplementary Table S1, and verified for fidelity by DNA sequencing in Beijing Genomics Institute (Beijing, China). The genes *ydhE* or *nhaD* were constructed into pEASY T3, together with their respective native promoters. The genome of *E. coli* was used for the template of *ydhE* encoding a H^+^-coupled MATE-MDR transporter of *E. coli* K12, whereas the previous construct, pUC-nhaD (Wang et al., 2017), was used for the template of *nhaD* encoding a Na^+^(Li^+^)/H^+^ antiporter from *H. alkaliphila* DSM16354^*T*^.

### Preparation of Everted Membrane Vesicles

*E. coli* CM2 or KNabc transformants were used to prepare the everted membrane vesicles by the French press method as described in our recent studies (Dong et al., 2017; Meng et al., 2017; Shao et al., 2018; Xu et al., 2019). Briefly, cultures were harvested and crushed by one passage under high pressure. Everted membrane vesicles were collected from the supernatant by supercentrifugation at 100,000 × *g* for 1 h after cell debris was removed. Then, everted membrane vesicles were resuspended and stored at −80°C.

### Na^+^/H^+^ Antiport Activity Assay

Na^+^/H^+^ antiport activity assay was performed using the everted membrane vesicles from *E. coli* KNabc transformants as described in our recent studies (Dong et al., 2017; Meng et al., 2017; Shao et al., 2018; Xu et al., 2019). A reaction buffer of indicated pH was made from 10 mM BTP/HCl, 140 mM choline chloride, and 250 mM sucrose. Before the fluorescent measurement, everted membrane vesicles containing ∼100 μg of total membrane protein and 1 μM acridine orange were added successively. Tris-D-lactate (10 mM) was added in order to create the quenching of acridine orange fluorescence by supplying the respiration. Antiport activity was represented by the dequenching ratio induced by the addition of NaCl at the final concentration of 5 mM. Fluorescence was measured with a fluorescence spectrophotometer F-7000 (Hitachi High-Technologies, Japan) with excitation at 492 nm and emission at 526 nm.

### Membrane Protein Expression Level Assay

Expression levels of MdrP or its variants were judged by Western blot using the everted membrane vesicles from *E. coli* CM2 transformants. Everted membrane vesicles with 10 μg proteins were subjected to 12% sodium dodecyl sulfate–polyacrylamide gel electrophoresis (SDS-PAGE) and electroblotted to nitrocellulose membranes. A rabbit anti-6 × His-tag antibody (Abcam Ltd., China) and a goat antirabbit secondary antibody (Nachuan Biotechnology Co., Ltd., Changchun, China) were used. Positive signals were probed by a horseradish peroxidase (Nachuan Biotechnology Co., Ltd., Changchun, China) via a Tanon-5200 multiautomatic chemiluminescence/fluorescence imaging system (Tanon Co., Ltd., China).

### Norfloxacin Accumulation Assay

Norfloxacin accumulation assay was conducted using the whole cells of *E. coli* CM2 transformants according to the modified protocol by Morita et al. (1998). Cells were grown to OD_600 *nm*_ of 1.0 in 100 ml of LB broth supplemented with 40 mM Tris-lactate instead of potassium lactate to create the transmembrane proton gradient and avoid K^+^ contamination. Cells were harvested by centrifugation, washed with a 100 mM Tris–HCl buffer (pH 7.0), and resuspended to OD_600 *nm*_ of 1.0 in the same buffer (∼60 ml). To initiate the assay, norfloxacin was added at the final concentration of 100 μM to the mixture. On each time point, 1 ml of cells were sampled in triplicate and centrifuged at 10,000 × *g* for 30 s at 4°C to stop the reaction. CCCP was added at the final concentration of 100 μM to the mixture to disrupt the transmembrane proton gradient. To analyze the effect of CCCP on intracellular accumulation of norfloxacin, cells were equally aliquoted after 20 min of incubation and continued to be incubated in the same buffer with or without the addition of CCCP. As the incubation time was increased, each sample without CCCP treatment was washed once with the same buffer, whereas that with CCCP treatment was washed once with the same buffer supplemented with 100 μM CCCP. Norfloxacin accumulated in the cells was completely extracted to the supernatant as follows. Each pellet was resuspended in 1 ml of 100 mM glycine–HCl buffer (pH 3.0) and shaken vigorously for 1 h at 25°C to release the fluorescent content and then centrifuged at 10,000 × *g* for 1 min. The supernatant was diluted by twofold in the 100 mM glycine–HCl buffer (pH 3.0), and then, the fluorescence of the supernatant was measured using a Hitachi F-7000 fluorescence spectrophotometer (Hitachi Ltd., Japan) at emission and excitation wavelengths of 488 and 227 nm, respectively. The amount of maximum fluorescence was normalized to 100% only when CCCP was added to the 100 mM Tris–HCl buffer (pH 7.0). The percentage ratio of measured fluorescence to maximum fluorescence was recorded to represent intracellular accumulated norfloxacin. To analyze the effect of NaCl on intracellular norfloxacin accumulation, cells were resuspended in the 100 mM Tris–HCl buffer (pH 7.0) supplemented with 100 mM NaCl. Then, the assay was initiated by the addition of norfloxacin, followed by the same procedure as the one described above.

### Efflux of Norfloxacin in the Presence of Na^+^

The assay for efflux of norfloxacin in the presence of Na^+^ was conducted using the whole cells of *E. coli* CM2 transformants. Cells were grown to OD_600 *nm*_ of 1.0 in 200 ml of LB broths at 37°C, harvested, and washed twice with a 100 mM Tris–HCl buffer (pH 7.0). To preload the cells with norfloxacin, cells were resuspended to OD_600 *nm*_ of 1.0 in the same buffer supplemented with 100 μM norfloxacin and 100 μM CCCP (∼170 ml) and continued to be incubated at 37°C for 30 min. After that, 1 ml of cells was sampled in triplicate on the time points of 5 and 10 min and then washed once with the same buffer supplemented with 100 μM CCCP. According to the preliminary experiments, intracellular accumulated norfloxacin reached the same maximum level within < 10 min. After 10 min of incubation, cell suspensions were equally aliquoted (30 ml for each), and NaCl was supplemented to each aliquot at the indicated concentrations, respectively. One milliliter of cells continued to be sampled in triplicate on the following time points and then washed once with the same buffer supplemented with 100 μM CCCP plus NaCl at the same concentrations as the ones used for the incubation. The intracellular accumulated norfloxacin was extracted and measured using the same method as the one described above.

### Measurement of Intracellular Na^+^ Contents

Cultures of *E. coli* KNabc transformants with pEASY T3-mdrP or pEASY T3 as the negative control were grown overnight and diluted 100-fold into 200 ml of fresh LBO broth. Cells were harvested at OD_600 *nm*_ of 1.0 and washed two times with a 100 mM Tris–HCl buffer (pH 7.5) containing 140 mM choline chloride and 0.2% glucose and then resuspended into 50 ml of the same buffer at 4°C. Cell suspensions were adjusted to the same total protein concentration and incubated at 25°C for 10 min before the reaction. To initiate the reaction, NaCl was added at the final concentration of 0.2 M to cell suspensions, unless indicated. To analyze the effect of norfloxacin on intracellular Na^+^ content, cell suspensions were supplemented with or without 2 μg/ml norfloxacin. On each time point, 3 ml of cell suspensions were sampled in triplicate, respectively. Samples were centrifuged at 10,000 × *g* for 30 s at 4°C and washed twice by the same ice-precooled buffer to terminate the reaction. Then, the pellets were resuspended in 2.5 ml of 5% trichloroacetic acid (TCA) and passed through the 0.22 μm polyethersulfone (PES) membrane to lyse the cells and dissolve the intracellular Na^+^. Intracellular Na^+^ contents were determined using an atomic absorption spectrophotometer AA-6650 (Shimadzu, Kyoto, Japan). To analyze the intracellular Na^+^ contents of *E. coli* KNabc expressing the variants of MdrP, cell suspensions were preincubated in the same buffer supplemented with 0.2 or 50 mM NaCl at 25°C for 60 min. The same steps of sample handling and measurement were followed as the one described above.

### Protein Concentration Determination

Protein concentrations were determined by the Bradford protein assays (Bradford, 1976), using bovine serum albumin (Tiangen Biotech, Co., Ltd., China) as a standard.

### Bioinformatic Analysis and Homology Structure Modeling

The protein alignment of MdrP with its 30 representative homologs clustered within our recently reported phylogenetic tree (Abdel-Motaal et al., 2018) was performed by DNAMAN 8.0, and the sequence logo was constructed through the submission of the aligned sequences by the software MEGA 5.0 to the WebLogo 3 website http://weblogo.threeplusone.com. The modeled structure of MdrP was constructed with *E. coli* YajR 3D structure (PDB ID 3WDO) (Jiang et al., 2013) and *Staphylococcus hominis* PepT (PDB ID 6EXS) (Minhas et al., 2018) as the respective templates of outward-facing conformation and inward-facing one by submitting the deduced amino acid sequence of MdrP to the Phyre2 website http://www.sbg.bio.ic.ac.uk/~phyre2/html/page.cgi?id=index, and produced using the PyMOL Molecular Graphics System (Schrödinger, LLC). The structural reliability was analyzed at the RAMPAGE website http://mordred.bioc.cam.ac.uk/~rapper/rampage.php.

## Results

### H^+^- and Na^+^-Coupled Norfloxacin Efflux Activity of MdrP

**FIGURE 1:**
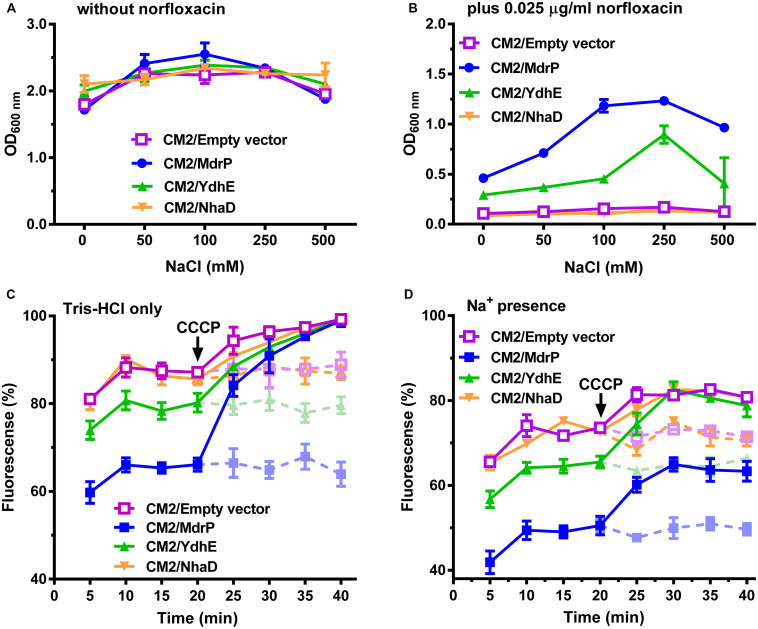
Norfloxacin resistance and efflux activity of MdrP dependent on H^+^ and Na^+^. Effects of NaCl concentrations on the growth of *E. coli* CM2 transformants were analyzed **(A)** in the absence of norfloxacin or **(B)** in the presence of 0.025 μg/ml norfloxacin. Precultures of *E. coli* (CM2/MdrP, blue filled circle), CM2/YdhE (green filled upward triangle), CM2/NhaD (brown filled downward triangle), or CM2/empty vector (purple open square) were grown in Luria–Bertani without the addition of NaCl (LBO) broths to OD_600 nm_ of 1.0. Then, 1% of the above precultures continued to be grown in LBO broths containing the indicated NaCl concentrations **(A)** without norfloxacin or **(B)** also supplemented with 0.025 μg/ml norfloxacin. Cell growth was ended within 24 h, followed by the evaluation of OD_600 nm_. Each data point stands for the mean ± SD of three independent cultures. Intracellular accumulation of norfloxacin in *E. coli* CM2 transformants was analyzed **(C)** in the absence of NaCl or **(D)** in the presence of 100 mM NaCl. Figure legends are the same ones as shown in **(A,B)**. Cells were incubated in a Tris–HCl buffer (pH 7.0) **(C)** without NaCl or **(D)** also supplemented with 100 mM NaCl. To initiate the assay, norfloxacin was added at the final concentration of 100 μM to the mixture. Cells were sampled on the indicated time points, and norfloxacin accumulated in the cells was completely extracted to the supernatant by incubating the sampled cells in a 100 mM glycine–HCl buffer (pH 3.0) for 1 h at 25°C. Carbonyl cyanide m-chlorophenylhydrazone (CCCP) was used at the final concentration of 100 μM to disrupt the transmembrane proton gradient. After 20 min (indicated by the downward arrows), solid and dotted lines represent the intracellular accumulation of norfloxacin in the above *E. coli* CM2 transformants with or without CCCP treatment, respectively. The intracellular norfloxacin contents were determined by measuring the fluorescence of norfloxacin at the excitation and emission wavelengths of 227 and 488 nm, respectively. Each data point stands for the mean ± SD of three independent measurements.

To test whether MdrP extrudes norfloxacin by coupling to H^+^ or Na^+^, the dependence of norfloxacin resistance of MdrP on H^+^ or Na^+^ was first analyzed by growing the transformants of *E. coli* CM2 (Δ*acrAB*), a major multidrug efflux system-deficient *E. coli* DH5α mutant (Abdel-Motaal et al., 2018), in LBO broths containing the indicated NaCl concentrations supplemented with or without 0.025 μg/ml norfloxacin. *E. coli* YdhE, a MATE-MDR transporter, has been identified to extrude multiple antimicrobial drugs including norfloxacin by the coupling of H^+^ translocation (Long et al., 2008). *Halomonas alkaliphila* NhaD has been identified by our lab to function as a Na^+^(Li^+^)/H^+^ antiporter (Wang et al., 2017). Therefore, pEASY T3-ydhE was used for the positive control of a H^+^-coupled norfloxacin efflux transporter, whereas pEASY T3-nhaD was used for the positive control of a Na^+^/H^+^ antiporter. As a preliminary test, the effect of NaCl concentrations on the growth of *E. coli* CM2 transformants was first analyzed in the absence of norfloxacin. There was no difference in growth between CM2/MdrP, CM2/YdhE, CM2/NhaD, and CM2/pEASY T3 in the absence of norfloxacin, and the growth of the above *E. coli* CM2 transformants remained almost unchanged as NaCl concentrations increased from 0 to 500 mM (Figure 1A). In contrast, a significant difference in growth was observed between the above *E. coli* CM2 transformants in the presence of 0.025 μg/ml norfloxacin as NaCl concentrations increased (Figure 1B). CM2/pEASY T3 did not grow in the presence of 0.025 μg/ml norfloxacin as NaCl concentrations increased (Figure 1B), which is consistent with the minimum inhibitory concentration (MIC) of *E. coli* CM2 for norfloxacin at 0.025 μg/ml (Abdel-Motaal et al., 2018). However, the growth of CM2/MdrP in the presence of 0.025 μg/ml norfloxacin was significantly enhanced as NaCl concentrations increased, especially at 100 mM or above (Figure 1B). In contrast, the growth of CM2/YdhE in the presence of 0.025 μg/ml norfloxacin was almost unaffected by the addition of NaCl at the different concentrations, except at 250 mM, as compared to that without NaCl (Figure 1B). Moreover, the growth curves of the above *E. coli* CM2 transformants were plotted in the presence of 0.025 μg/ml norfloxacin without NaCl or with the addition of 250 mM NaCl (Supplementary Figure S1). Under the tested conditions, CM2/pEASY T3 or CM2/NhaD did not grow, whereas CM2/MdrP or CM2/YdhE could grow to the stationary phase within 24 h (Supplementary Figures S1A,B). This reveals that the evaluation of OD_600 *nm*_ on 24 h can be used to represent the growth of the above *E. coli* CM2 transformants under the tested conditions. Notably, the growth of CM2/MdrP was higher by one to threefold than that of CM2/YdhE under the same tested conditions, even in the presence of 0.025 μg/ml norfloxacin without NaCl. Since YdhE is an identified H^+^-coupled norfloxacin efflux transporter (Long et al., 2008), it is very likely that MdrP may function at least as a H^+^-coupled norfloxacin efflux transporter. Considering that norfloxacin resistance of MdrP was significantly stimulated by the addition of NaCl (Figure 1B), MdrP may also function as a Na^+^-coupled norfloxacin efflux transporter. CM2/NhaD showed the same growth with that of CM2/pEASY T3 under all the tested conditions (Figures 1A,B). This ruled out the possibility that Na^+^/H^+^ antiport activity of MdrP affected the proton motive force and thus offered the Na^+^-stimulated norfloxacin resistance.

To establish that efflux of norfloxacin by MdrP may be H^+^ coupled, norfloxacin accumulation was first assayed using the cells of *E. coli* CM2 transformants incubated only in a 100 mM Tris–HCl buffer. As compared with CM2/pEASY T3, CM2/MdrP showed significantly lower intracellular norfloxacin accumulation than that of CM2/YdhE as the incubation time was increased. Such difference in accumulation of norfloxacin between them was completely eradicated by the addition of CCCP (Figure 1C). This reveals that MdrP functions as a H^+^-coupled norfloxacin efflux transporter in a mode of drug/H^+^ antiport, just as YdhE. To test whether efflux of norfloxacin by MdrP may also be Na^+^-coupled, norfloxacin accumulation was further assayed using the cells of *E. coli* CM2 transformants incubated in the 100 mM Tris–HCl buffer supplemented with 100 mM NaCl. The difference in accumulation of norfloxacin between CM2/YdhE and CM2/pEASY T3 was completely removed by the addition of CCCP even in the presence of 100 mM NaCl (Figure 1D). This confirms that YdhE only functions as a H^+^-coupled norfloxacin efflux transporter. In contrast, the difference in accumulation of norfloxacin between CM2/MdrP and CM2/pEASY T3 was not removed by the addition of CCCP in the presence of 100 mM NaCl (Figure 1D). However, such difference in accumulation of norfloxacin between them was exactly removed by the addition of CCCP only in the 100 mM Tris–HCl buffer (Figure 1C). This suggests that MdrP may remain the partial or intact norfloxacin efflux activity after the addition of CCCP in the presence of Na^+^. Therefore, it is very likely that MdrP can function as a Na^+^-coupled norfloxacin efflux transporter. CM2/NhaD showed the same intracellular norfloxacin accumulation with that of CM2/pEASY T3 in the absence or presence of Na^+^ (Figures 1C,D). This ruled out the possibility that Na^+^/H^+^ antiport activity of MdrP affected the proton motive force and thus led to the difference in accumulation of norfloxacin between CM2/MdrP and CM2/pEASY T3. Accumulation of norfloxacin in all the *E. coli* CM2 transformants was reduced ∼20% in the presence of 100 mM NaCl (Figure 1D) as compared to that in the absence of NaCl (Figure 1C), with or without the addition of CCCP. This may be attributed to the stimulatory effect of Na^+^ on the activity of potential Na^+^-coupled norfloxacin efflux transporters of host strain.

**FIGURE 2:**
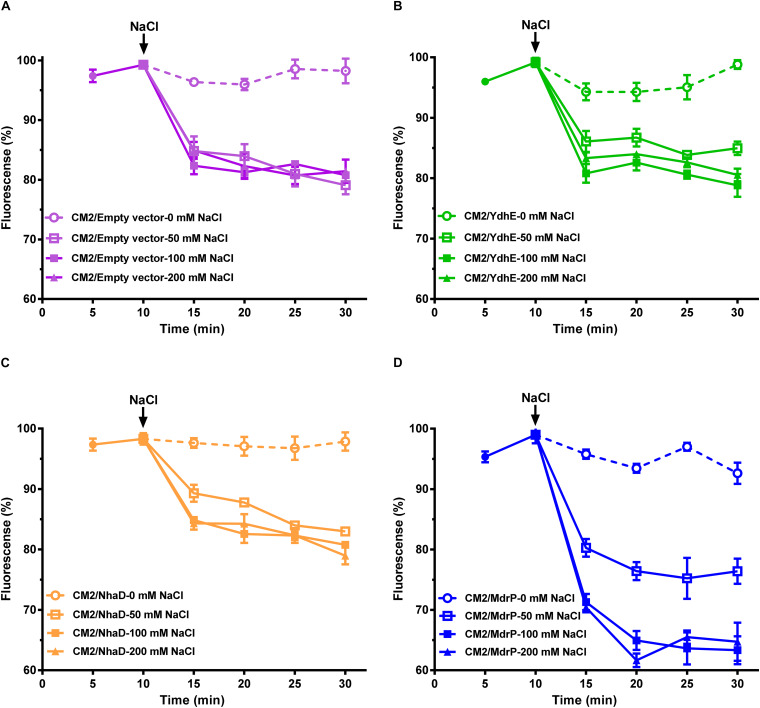
Efflux of norfloxacin by MdrP in the presence of Na^+^. The assay for efflux of norfloxacin in the presence of Na^+^ was conducted using the whole cells of *E. coli* CM2 transformants. **(A–D)** shows the effects of NaCl concentrations on efflux of norfloxacin accumulated in *E. coli* CM2/pEASY T3 (in purple), CM2/YdhE (in green), CM2/NhaD (in brown), and CM2/MdrP (in blue), respectively. Open circle stands for intracellular accumulation of norfloxacin in the above *E. coli* CM2 transformants measured at 0 mM NaCl; open diamond stands for the one measured at 50 mM NaCl; open square stands for the one measured at 100 mM NaCl; open square stands for the one measured at 200 mM NaCl. The incubations of the above *E. coli* CM2 transformants were kept to be performed under the control of 100 μM carbonyl cyanide m-chlorophenylhydrazone (CCCP) during the whole reaction. Cells were preincubated for 30 min in a Tris–HCl buffer (pH 7.0) supplemented with 100 μM norfloxacin and 100 μM CCCP to preload cells with norfloxacin. After that, 1 ml of cells was sampled in triplicate on the time points of 5 and 10 min to test whether the intracellular norfloxacin accumulation reached the maximum level (indicated by the downward arrows). Then, cell suspensions were divided into four equal aliquots (30 ml for each), and NaCl was supplemented to each aliquot at the indicated concentrations, respectively. One milliliter of cells continued to be sampled in triplicate on the following time points and then washed once with the same buffer supplemented with 100 μM CCCP plus NaCl at the same concentrations as those used for the incubation. Norfloxacin accumulated in the cells was completely extracted to the supernatant by incubating the sampled cells in a 100 mM glycine–HCl buffer (pH 3.0) for 1 h at 25°C. The intracellular norfloxacin contents were determined by measuring the fluorescence of norfloxacin at the excitation and emission wavelengths of 227 and 488 nm, respectively. Each data point stands for the mean ± SD of three independent measurements.

The above results presented in Figures 1C,D enlighten us that Na^+^-coupled norfloxacin efflux activity can be detected only when the transmembrane proton gradient is disrupted by the addition of CCCP. That is to say, after intracellular norfloxacin is accumulated to the maximum level by the addition of CCCP in the absence of NaCl, if accumulation of norfloxacin in the tested cells can be reduced by the addition of NaCl, the reduced norfloxacin should be caused only by Na^+^-coupled norfloxacin efflux activity. Thus, Na^+^-coupled norfloxacin efflux activity was assayed by analyzing the effect of NaCl concentrations on the reduction in norfloxacin accumulated in *E. coli* CM2 transformants. The accumulation of norfloxacin in all the *E. coli* CM2 transformants reached the same maximum level after the addition of CCCP in the absence of NaCl and remained almost unchanged as the incubation time was increased (Figures 2A–D). This reveals that norfloxacin/H^+^ antiport activity or/and Na^+^/H^+^ antiport activity of NhaD, YdhE, MdrP, or host strain cannot affect accumulation of norfloxacin in the tested cells in the presence of CCCP without NaCl. In contrast, the intracellular norfloxacin accumulated in CM2/pEASY T3 (Figure 2A), CM2/YdhE (Figure 2B), and CM2/NhaD (Figure 2C) was reduced to almost the same level as NaCl concentrations increased from 50 to 200 mM. This may be attributed to the stimulatory effect of Na^+^- on Na^+^-coupled norfloxacin efflux activity of host strain, since the transmembrane proton gradient was disrupted by the addition of CCCP. Importantly, the intracellular norfloxacin accumulated in CM2/MdrP was significantly reduced to the lower level than those of three controls as NaCl concentrations increased, especially at 100–200 mM (Figure 2D). Therefore, MdrP could exactly function as a Na^+^-coupled norfloxacin efflux transporter.

### Function of MdrP in a Mode of Drug/Na^+^ Antiport

**FIGURE 3:**
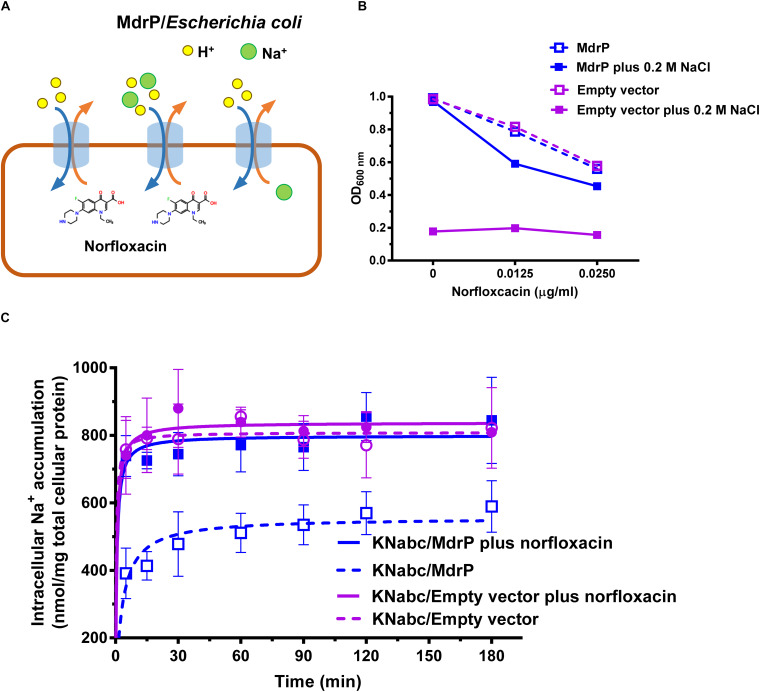
Function of MdrP as a drug/Na^+^ antiporter. **(A)** Schematic representation of the proposed MdrP antiport mechanism. MdrP is drawn as a blue cylinder; Na^+^ is drawn as a green filled circle; H^+^ is drawn as a yellow circle; norfloxacin is represented by its 2D structure formula. Brown and blue curve arrows in the opposite direction stand for the antiport of norfloxacin/Na^+^(H^+^) and Na^+^/H^+^. **(B)** Effect of norfloxacin concentrations on the growth of *E. coli* KNabc transformants in Luria–Bertani without the addition of NaCl (LBO) broths with the indicated norfloxacin concentrations supplemented with or without 0.2 M NaCl. Purple open circle with purple dotted line stands for the growth of KNabc/pEASY T3 (KNabc/empty vector) in the absence of NaCl; blue open circle with blue dotted line stands for the growth of KNabc/MdrP in the absence of NaCl; purple filled square with purple solid line stands for KNabc/Empty vector in the presence of 0.2 M NaCl; blue filled square with blue solid line stands for KNabc/MdrP in the presence of 0.2 M NaCl. Cell growth was ended within 48 h, followed by the evaluation of OD_600 nm_. To better assess whether the presence of Na^+^ affected cell growth, the data were presented as the relative growth ratios (ratio of tested OD_600 nm_ to the maximum OD_600 nm_ in the absence of norfloxacin). Each data point stands for the mean ± SD of three independent cultures. **(C)** Intracellular Na^+^ contents of *E. coli* KNabc transformants in the presence of 0.2 M NaCl with or without the addition of norfloxacin. Figure legends are the same ones as shown in **(B)**. For the analysis of the intracellular Na^+^ contents, *E. coli* KNabc transformants were grown in LBO broths to OD_600 nm_ of 1.0, and aliquots of cells were incubated in a 100 mM Tris–HCl buffer (pH 7.5) containing 140 mM choline chloride and 0.2% glucose, plus 200 mM NaCl. Immediately when the incubation was started, 2 μg/ml norfloxacin was added to test the effect of norfloxacin on intracellular Na^+^ contents. The intracellular Na^+^ contents were determined using an atomic absorption spectrophotometer AA-6650 (Shimadzu, Kyoto, Japan). Each data point stands for the mean ± SD of three independent measurements.

The above results establish that MdrP can function as a drug/H^+^ antiporter independently of Na^+^ (Figure 3A), and efflux of norfloxacin by MdrP is also Na^+^ coupled. However, it needs to be confirmed whether the coupling of Na^+^ to norfloxacin efflux operates in a drug/Na^+^ antiport mode (Figure 3A). Here, a major Na^+^/H^+^ antiporter-deficient *E. coli* mutant KNabc (Nozaki et al., 1996) was used to address this question. The mutant transformed with the empty vector, pEASY T3, did not grow in the presence of 0.2 M NaCl as norfloxacin concentrations increased from 0 to 0.025 μg/ml (Figure 3B), due to the deficiency of three major Na^+^/H^+^ antiporters, NhaA, NhaB, and ChaA (Nozaki et al., 1996). As for drug resistance, *E. coli* KNabc is equivalent to a wild-type strain of *E. coli* because it has a major drug efflux system, AcrAB. Therefore, KNabc/MdrP or KNabc/pEASY T3 showed the same reduction trend in growth in the absence of NaCl, as norfloxacin concentrations increased (Figure 3B). Being a Na^+^/H^+^ antiporter (Figure 3A), MdrP offered the tolerance of *E. coli* KNabc to 0.2 M NaCl in the absence or presence of norfloxacin (Figure 3B). If MdrP did not function as a norfloxacin/Na^+^ antiporter, the growth of KNabc/MdrP in the presence of 0.2 M NaCl would show the same negative linear correlationship with that in the absence of NaCl as norfloxacin concentrations increased. However, the growth of KNabc/MdrP was more reduced in the presence of 0.2 M NaCl than that in the absence of NaCl, as norfloxacin concentrations increased (Figure 3B). Therefore, MdrP would very likely bring an extra amount of Na^+^ into the cells of *E. coli* KNabc while it extruded norfloxacin outside the cells. To confirm that efflux of norfloxacin by MdrP is coupled with Na^+^ influx, the intracellular Na^+^ contents in KNabc/MdrP were analyzed in the presence of 0.2 M NaCl with or without the addition of norfloxacin, using KNabc/pEASY T3 as a negative control. KNabc/MdrP showed significantly lower intracellular Na^+^ content in the absence of norfloxacin than that of KNabc/pEASY T3 with or without norfloxacin treatment (Figure 3C), which reveals that MdrP can reduce the intracellular Na^+^ content in *E. coli* KNabc via its Na^+^/H^+^ antiport activity (Figure 3A). In contrast, addition of norfloxacin significantly enhanced the intracellular Na^+^ content in KNabc/MdrP, which reached the same level with those in KNabc/pEASY T3 (Figure 3C). This establishes the fact that efflux of norfloxacin by MdrP led to influx of Na^+^ into the cells of *E. coli* KNabc. Moreover, it is possible that Na^+^ concentration was increased in the presence of norfloxacin by changing the preference to transport norfloxacin over Na^+^ while importing H^+^ into the cell (Figure 3C). However, this possibility can be ruled out based on the results presented in Figures 1B,D, 2D, 3B. For example, if MdrP did not function as a drug/Na^+^ antiporter, it would be impossible that the norfloxacin efflux activity of MdrP were detected in the presence of CCCP (Figures 1D, 2D). Therefore, MdrP could function as a drug/Na^+^ antiporter.

### Selection of Function- or Structure-Related Residue Candidates

Sequence conservation was first considered to predict function- or structure-related residue candidates of MdrP. However, MdrP was an uncharacterized MFS member that shares relatively low identity with the two phylogenetically closest identified homologs, *L. lactis* LmrP and *E. coli* MdtH (Abdel-Motaal et al., 2018). As a result, there are not enough available identified homologs of MdrP to be applicable for protein alignment. However, our recent study showed that MdrP exhibited the phylogenetically close relationship with 30 representatives of its putative homologs at a wide identity range from 29% to 95% (Abdel-Motaal et al., 2018). This suggests that they may have been evolved from the same ancestor. Therefore, these 30 representative homologs were used to create the protein alignment with MdrP for the residue conservation analysis (Supplementary Figure S2). Within the identity range from 29% to 95%, five acidic residues (D67, D127, E188, D223, and E341) and one alkaline residue (R71) are fully conserved (Supplementary Figure S2), and one acidic residue (D244) and one alkaline residue (R361) are highly conserved (Supplementary Figure S2).

To accurately predict the roles of residues, the modeled structure of MdrP with 100% confidence succeeded in being constructed using the structures of *E. coli* YajR 3D (PDB ID 3WDO) and *Staphylococcus hominis* PepT (PDB ID 6EXS) as the respective templates of outward- and inward-facing conformations (Supplementary Figures S3A–D). MdrP shares full-length coverage and 16 and 20% identities with YajR and PepT, respectively (Supplementary Figure S3G). The sole four residues S99, T254, P357, and A379 fall in the outlier region of Ramachandran plot of outward-facing modeled structure (Supplementary Figure S3E), whereas the sole residue D223 falls in the outlier region of Ramachandran plot of inward-facing modeled structure (Supplementary Figure S3F). This reveals that both modeled structures of MdrP are reliable and appropriate for the prediction of residue roles based on their locations.

**FIGURE 4:**
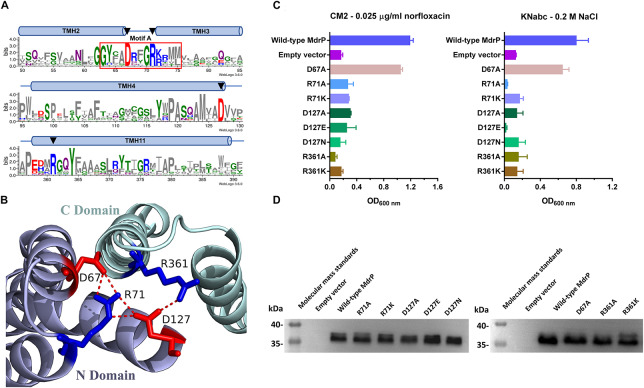
Selection of conformation-related residues and the effect of each variant on norfloxacin resistance. **(A)** Four potential conformation-related residues (D67, R71, D127, and R361) were selected for site-directed mutagenesis on the basis of the residue conservation analysis by constructing a weblogo of MdrP and 30 representatives of its homologs (Abdel-Motaal et al., 2018) and the **(B)** prediction of polar contacts between residues in a modeled structure. **(C)** Norfloxacin resistance and NaCl tolerance of each variant was tested in triplicate by its complementation with *E. coli* CM2 in LB broths containing 0.025 μg/ml norfloxacin or the one with *E. coli* KNabc in Luria–Bertani with NaCl replaced by 89 mM KCl (LBK) broths containing 0.2 M NaCl. Wild-type MdrP was used as a positive control, whereas the empty vector pEASY T3 was used as a negative control. Precultures of *E. coli* CM2 or KNabc transformants were grown in LB (for CM2) or LBK (for KNabc) broths to OD_600 nm_ of 1.0. Then, 1% of the above precultures continued to be grown in LB broths supplemented with 0.025 μg/ml norfloxacin or LBK broths containing 0.2 M NaCl. Cell growth was ended within 24 h for CM2 and 48 h for KNabc, followed by the evaluation of OD_600 nm_. Each data point stands for the mean ± SD of three independent cultures. **(D)** Expression level of each variant was analyzed using membrane fractions from the corresponding *E. coli* CM2 transformant.

In the modeled structures of MdrP, TMH1-6 and TMH7-12 constitute the N and C domains, respectively, and both of them form an outward- or inward-facing central cavity in the transmembrane core (Supplementary Figures S3A–D) as is typical in the structures of the majority of MFS transporters (Yan, 2013a). D67 and R71 are located in motif A between TMH2 and TMH3, and D127 is located in the C terminus of TMH4 (Figure 4A and Supplementary Figure S2), and E188 is located in the loop between TMH6 and TMH7 (Supplementary Figure S2). These four residues are located at the N domain-membrane interface on the cytoplasmic side (Supplementary Figures S3A,C). D244 is located in the middle of TMH7, E341 is located in the middle of TMH10, D223 is located in the unwound segment between helix α 6–7 and TMH7, and R361 is adjacent to the N terminus of TMH11 (Supplementary Figure S2). The latter four residues belong to C domain of MdrP, of which D244 and E341 face the central cavity, whereas D223 is located in the C domain-membrane interface on the cytoplasmic side (Supplementary Figure S3A). In *E. coli* YajR, D73 and R77 in motif A and D126 in TMH4 have been documented to play an important role in the stabilization of the outward-facing conformation (Jiang et al., 2013). Notably, D67, R71, and D127 of MdrP corresponds to D73, R77, and D126 of YajR (Supplementary Figure S3G), respectively. In addition, D67, R71, and D127 were predicted to form polar contacts with R361 at the interface of N, C domains on the cytoplasmic side (Figure 4B and Supplementary Figures 3A,C).

On the basis of sequence conservation, residue locations, and possible polar contacts between residues, the above six acidic residues and two alkaline residues were selected to analyze their relationship with the function and structure of MdrP.

### Involvement of R71, D127, and R361 in the Conformational Stabilization of MdrP

**FIGURE 5:**
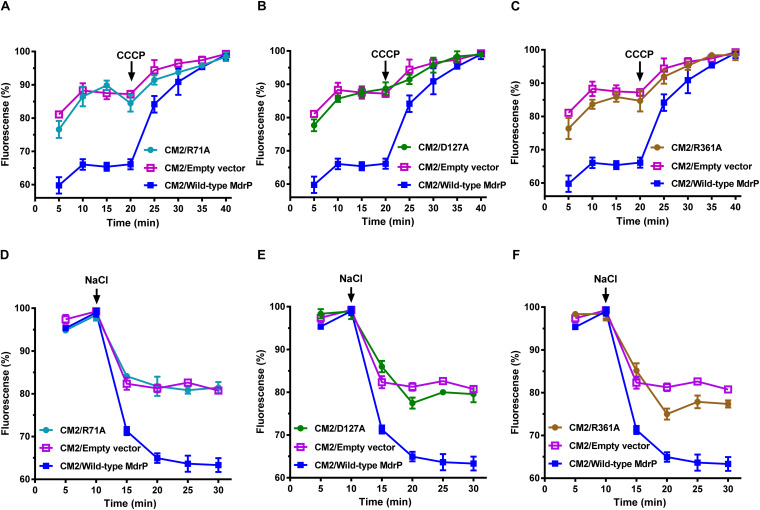
Determination of H^+^- and Na^+^-coupled norfloxacin efflux activity of R71A, D127A, and R361A. **(A–C)** Intracellular accumulation of norfloxacin in *E. coli* CM2 expressing R71A (cyan filled circle), D127A (green filled circle), or R361A (yellow filled circle) was analyzed in the absence of NaCl. To initiate the assay, norfloxacin was added at the final concentration of 100 μM to the mixture. After 20 min (indicated by the downward arrows), carbonyl cyanide m-chlorophenylhydrazone (CCCP) was used at the final concentration of 100 μM to disrupt the transmembrane proton gradient. **(D–F)** Efflux of norfloxacin accumulated in *E. coli* CM2 expressing R71A (cyan filled circle), D127A (green filled circle), or R361A (yellow filled circle) was analyzed in the presence of 100 mM NaCl. The incubations of the above *E. coli* CM2 transformants were kept to be performed under the control of 100 μM CCCP during the whole reaction. Cells were preincubated for 30 min in a Tris–HCl buffer (pH 7.0) supplemented with 100 μM norfloxacin and 100 μM CCCP to preload cells with norfloxacin. After that, 1 ml of cells was sampled in triplicate on the time points of 5 and 10 min to test whether the intracellular norfloxacin accumulation reached the maximum level. On the time point (indicated by the downward arrows), NaCl was supplemented to cell suspensions at the final concentration of 100 mM. **(A–F)** During the above activity assays, wild-type MdrP (blue filled square) was used as a positive control, whereas the empty vector pEASY T3 was used as a negative control (open purple square). Norfloxacin accumulated in the cells was completely extracted to the supernatant by incubating the sampled cells in a 100 mM glycine–HCl buffer (pH 3.0) for 1 h at 25°C. The intracellular norfloxacin contents were determined by measuring the fluorescence of norfloxacin at the excitation and emission wavelengths of 227 and 488 nm, respectively. Each data point stands for the mean ± SD of three independent measurements.

To test whether D67, R71, D127, and R361 are involved in the conformational stabilization of MdrP, each residue was substituted by alanine or other residues via site-directed mutagenesis, followed by the growth tests and norfloxacin accumulation assays. R71A, D127A, or R361A, even R71K, R361K, D127E, or D127N, did not offer the resistance of *E. coli* CM2 to 0.025 μg/ml norfloxacin (Figure 4C, left panel) or the tolerance of *E. coli* KNabc to 0.2 M NaCl (Figure 4C, right panel). However, D67A almost had no effect on the complementation with *E. coli* CM2 in the presence of 0.025 μg/ml norfloxacin (Figure 4C, left panel) or the one with *E. coli* KNabc in the presence of 0.2 M NaCl (Figure 4C, right panel). The above variants were shown to be expressed in *E. coli* CM2 as wild-type MdrP (Figure 4D). These results reveal that R71, D127, and R361 are indispensable for both activities of norfloxacin efflux and Na^+^/H^+^ antiport. Especially, both non-charged amino groups and positive charges of guanidyl groups are vital for the roles of R71 and R361 in both activities. In addition, both negative charge and the length of side chain are vital for the role of D127 in both activities. The above results support the existence of polar contacts between R71, D127, and R361 for the conformational stabilization of MdrP. To confirm this point, R71A, D127A, and R361A were selected to analyze the intracellular accumulation of norfloxacin in *E. coli* CM2 expressing each variant in the absence (Figures 5A–C) or presence (Figures 5D–F) of Na^+^, using wild-type MdrP as a positive control and the empty vector as a negative control. As expected, *E. coli* CM2 expressing R71A, D127A, or R361A showed completely or almost the same intracellular norfloxacin accumulation as the one in CM2 with the empty vector in the absence of Na^+^ (Figures 5A–C), when *E. coli* CM2 expressing wild-type MdrP showed significantly lower intracellular norfloxacin accumulation under the same condition (Figures 5A–C). The same results were also obtained in the presence of Na^+^ (Figures 5D–F). Therefore, substitution of each residue by alanine completely or almost resulted in the loss of both H^+^- and Na^+^-coupled norfloxacin efflux activity. The above results suggest that R71, D127, and R361 may be involved in the conformational stabilization of MdrP via polar contacts between them.

### Vital Roles of D223 and D244 in the Function of MdrP

**FIGURE 6:**
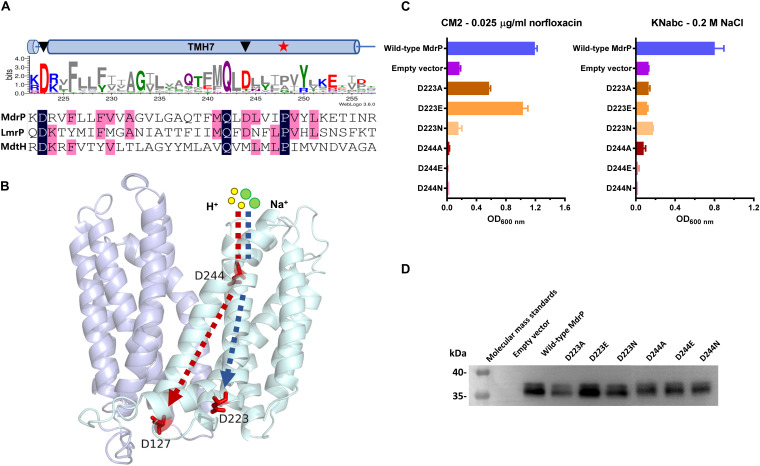
Selection of Na^+^- and H^+^-translocating residues and the effect of each variant on norfloxacin resistance. **(A)** Two potential Na^+^- and H^+^-translocating residues (D223 and D244) were selected for site-directed mutagenesis on the basis of the residue conservation analysis by constructing a weblogo (upper) of MdrP and 30 representatives of its homologs (Abdel-Motaal et al., 2018) combined with the protein alignment (lower) of MdrP with LmrP and MdtH and **(B)** the residue locations in a modeled structure. **(C)** Norfloxacin resistance and NaCl tolerance of each variant was tested in triplicate by its complementation with *E. coli* CM2 in Luria–Bertani (LB) broths containing 0.025 μg/ml norfloxacin or the one with *E. coli* KNabc in LB with NaCl replaced by 89 mM KCl (LBK) broths containing 0.2 M NaCl, using wild-type MdrP as a positive control and the empty vector pEASY T3 as a negative control. Precultures of *E. coli* CM2 or KNabc transformants were grown in LB (for CM2) or LBK (for KNabc) broths to OD_600 nm_ of 1.0. Then, 1% of the above precultures continued to be grown in LB broths supplemented with 0.025 μg/ml norfloxacin or LBK broths containing 0.2 M NaCl. Cell growth was ended within 24 h for CM2 and 48 h for KNabc, followed by the evaluation of OD_600 nm_. Each data point stands for the mean ± SD of three independent cultures. **(D)** The expression level of each variant was analyzed using membrane fractions from the corresponding *E. coli* CM2 transformant.

In addition to the conservation between MdrP and its representative homologs (Figure 6A, the upper panel; Supplementary Figure S2) and the important locations in the modeled structures (Figure 6B and Supplementary Figures S3A,B), two acidic residues, D223 and D244, also exhibit the full or partial conservation between MdrP and the two phylogenetically closest identified homologs with low identity, *L. lactis* LmrP and *E. coli* MdtH (Figure 6A, the lower panel). Therefore, D223 and D244 may be vital for the dual functions of MdrP as a Na^+^/H^+^ antiporter and also as a Na^+^- or/and H^+^-coupled norfloxacin efflux transporter. To establish this point, the complementation capability of the variants of D223 and D244 with *E. coli* CM2 and KNabc were first analyzed under the tested stress.

D223A partially offered the resistance of *E. coli* CM2 to 0.025 μg/ml norfloxacin (Figure 6C, left panel) but did not offer the tolerance of *E. coli* KNabc to 0.2 M NaCl (Figure 6C, right panel), suggesting that D223 may directly participate in Na^+^ and H^+^ translocation, rather than norfloxacin efflux. Substitution of D223 by glutamic acid solely restored the normal norfloxacin resistance of MdrP, whereas substitution of this residue by asparagine simultaneously abolished both norfloxacin resistance and NaCl tolerance of MdrP (Figure 6C), suggesting that the negative charge of side chain from D223 may satisfy the requirement for Na^+^ or/and H^+^ translocation coupled to norfloxacin efflux, whereas both negative charge and the length of side chain are the prerequisite for Na^+^/H^+^ antiport. In contrast, substitution of D244 by alanine, glutamic acid, or asparagine simultaneously abolished both norfloxacin resistance and NaCl tolerance of MdrP (Figure 6C), suggesting that both negative charge and the length of side chain from D244 may play a vital role in Na^+^/H^+^ antiport and Na^+^ and H^+^ translocation coupled to norfloxacin efflux. The above variants were shown to be expressed as wild-type MdrP in *E. coli* CM2 (Figure 6D).

### Simultaneous Participation of D244 in H^+^ and Na^+^ Translocation

**FIGURE 7:**
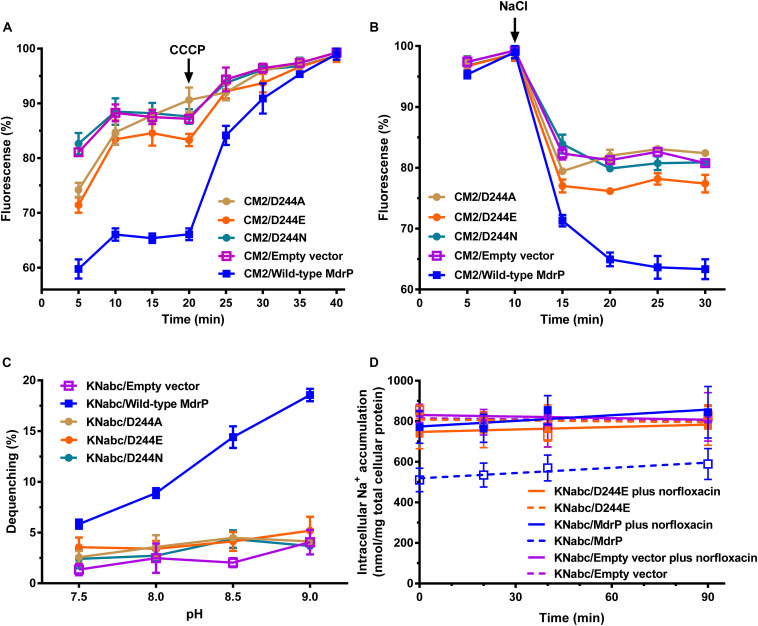
Determination of the role of D244 in H^+^ and Na^+^ translocation of MdrP. **(A)** Intracellular accumulation of norfloxacin in *E. coli* CM2 expressing each variant of D244 was analyzed in the absence of NaCl, using wild-type MdrP as a positive control and the empty vector pEASY T3 as a negative control. To initiate the assay, norfloxacin was added at the final concentration of 100 μM to the mixture. After 20 min (indicated by the downward arrows), carbonyl cyanide m-chlorophenylhydrazone (CCCP) was used at the final concentration of 100 μM to disrupt the transmembrane proton gradient. **(B)** Efflux of norfloxacin accumulated in *E. coli* CM2 expressing each variant of D244 was analyzed in the presence of 100 mM NaCl, using wild-type MdrP as a positive control and the empty vector pEASY T3 as a negative control. The incubations of the above *E. coli* CM2 transformants were kept to be performed under the control of 100 μM CCCP during the whole reaction. Cells were preincubated for 30 min in a Tris–HCl buffer (pH 7.0) supplemented with 100 μM norfloxacin and 100 μM CCCP to preload cells with norfloxacin. After that, 1 ml of cells was sampled in triplicate on the time points of 5 and 10 min to test whether the intracellular norfloxacin accumulation reached the maximum level. On the time point (indicated by the downward arrows), NaCl was supplemented to cell suspensions at the final concentration of 100 mM. **(A,B)** During the above activity assays, norfloxacin accumulated in the cells was completely extracted to the supernatant by incubating the sampled cells in a 100 mM glycine–HCl buffer (pH 3.0) for 1 h at 25°C. The intracellular norfloxacin contents were determined by measuring the fluorescence of norfloxacin at the excitation and emission wavelengths of 227 and 488 nm, respectively. Each data point stands for the mean ± SD of three independent measurements. **(C)** Na^+^/H^+^ antiport activity was determined using the everted membrane vesicles from *E. coli* KNabc expressing each variant of D244, using wild-type MdrP as a positive control and the empty vector pEASY T3 as a negative control. Each data point stands for the mean ± SD of three independent measurements. **(D)** Intracellular Na^+^ contents of *E. coli* KNabc/D244E were measured in the presence of 0.2 M NaCl with or without the addition of norfloxacin, using wild-type MdrP as a positive control and the empty vector pEASY T3 as a negative control. *E. coli* KNabc transformants were grown in Luria–Bertani without the addition of NaCl (LBO) broths to OD_600 nm_ of 1.0, and aliquots of cells were preincubated in the presence of 200 mM NaCl within 60 min. At that time, intracellular Na^+^ contents reached the steady level. Then, 2 μg/ml norfloxacin was added to test the effect of norfloxacin on the intracellular Na^+^ contents. The intracellular Na^+^ contents were determined using an atomic absorption spectrophotometer AA-6650 (Shimadzu, Kyoto, Japan). Each data point stands for the mean ± SD of three independent measurements.

To confirm that D244 participates in H^+^ and Na^+^ translocation, we analyzed the Na^+^- or H^+^-coupled norfloxacin efflux activity and Na^+^/H^+^ antiport activity of D244A, D244E, or D244N, using wild-type MdrP as a positive control and the empty vector as a negative control. *E. coli* CM2 transformants were assayed for norfloxacin efflux activity in the absence or presence of Na^+^. Substitution of D244 by alanine, glutamic acid, or asparagine was found to almost or completely abolish both H^+^- and Na^+^-coupled norfloxacin efflux activity of MdrP (Figures 7A,B). *E. coli* KNabc transformants were assayed for Na^+^/H^+^ antiport activity. Substitution of D244 by alanine, glutamic acid, or asparagine also found to abolish Na^+^/H^+^ antiport activity of MdrP (Figure 7C). D244E was further selected to analyze the effect of norfloxacin treatment on intracellular Na^+^ contents of KNabc/D224E in the presence of 0.2 M NaCl, using wild-type MdrP as a positive control and the empty vector as a negative control. Judging from the results presented in Figure 3C, the intracellular Na^+^ contents of KNabc/MdrP or KNabc/empty vector could reach the balanced maximum levels 60 min after incubation in the presence of 0.2 M NaCl. Therefore, the intracellular Na^+^ contents of *E. coli* KNabc transformants expressing the variants of MdrP started to be monitored after the 60 min preincubation at the tested NaCl concentrations in the current and following assays. Thus, when intracellular Na^+^ contents reached the balanced level, norfloxacin/Na^+^ antiport activity would be detected more easily by analyzing the effect of norfloxacin treatment on the balanced intracellular Na^+^ contents of *E. coli* KNabc transformants. KNabc/MdrP showed significantly lower intracellular Na^+^ content in the absence of norfloxacin, and its intracellular Na^+^ content was accumulated by the addition of norfloxacin to the same level as that of KNabc/empty vector with or without norfloxacin treatment (Figure 7D). However, KNabc/D244E accumulated the same intracellular Na^+^ content as that of KNabc/empty vector, with or without the addition of norfloxacin (Figure 7D). Since D244E lost both Na^+^/H^+^ antiport activity (Figure 7C) and drug/Na^+^ antiport activity (Figure 7B), it is reasonable that D244E could not change the intracellular Na^+^ content of *E. coli* KNabc with or without norfloxacin treatment (Figure 7D). The above results establish that D244 may simultaneously participate in H^+^ and Na^+^ translocation, which is responsible for not only Na^+^/H^+^ antiport but also H^+^ and Na^+^ coupling to norfloxacin efflux.

### Function of D223 as a Key Determinant in Na^+^ Translocation

**FIGURE 8:**
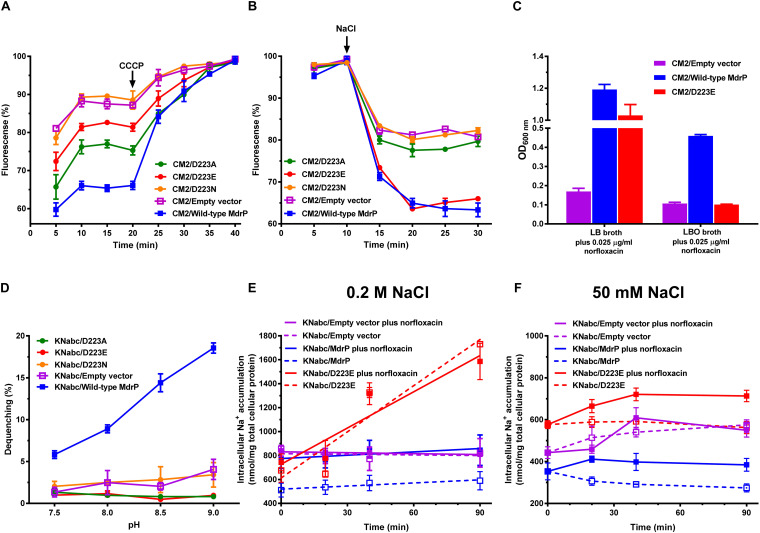
Determination of the role of D223 in H^+^ and Na^+^ translocation of MdrP. **(A)** Intracellular accumulation of norfloxacin in *E. coli* CM2 expressing each variant of D223 was analyzed in the absence of NaCl, using wild-type MdrP as a positive control and the empty vector pEASY T3 as a negative control. To initiate the assay, norfloxacin was added at the final concentration of 100 μM to the mixture. After 20 min (indicated by the downward arrows), carbonyl cyanide m-chlorophenylhydrazone (CCCP) was used at the final concentration of 100 μM to disrupt the transmembrane proton gradient. **(B)** Efflux of norfloxacin accumulated in *E. coli* CM2 expressing each variant of D223 was analyzed in the presence of 100 mM NaCl, using wild-type MdrP as a positive control and the empty vector pEASY T3 as a negative control. The incubations of the above *E. coli* CM2 transformants were kept to be performed under the control of 100 μM CCCP during the whole reaction. Cells were preincubated for 30 min in a Tris–HCl buffer (pH 7.0) supplemented with 100 μM norfloxacin and 100 μM CCCP to preload cells with norfloxacin. After that, 1 ml of cells were sampled in triplicate on the time points of 5 and 10 min to test whether the intracellular norfloxacin accumulation reached the maximum level. On the time point (indicated by the downward arrows), NaCl was supplemented to cell suspensions at the final concentration of 100 mM. **(A,B)** During the above activity assays, norfloxacin accumulated in the cells was completely extracted to the supernatant by incubating the sampled cells in a 100 mM glycine–HCl buffer (pH 3.0) for 1 h at 25°C. The intracellular norfloxacin contents were determined by measuring the fluorescence of norfloxacin at the excitation and emission wavelengths of 227 and 488 nm, respectively. Each data point stands for the mean ± SD of three independent measurements. **(C)** Growth of *E. coli* CM2/D223E in LB or Luria–Bertani without the addition of NaCl (LBO) broths supplemented with 0.025 μg/ml norfloxacin. Wild-type MdrP was used as a positive control, whereas the empty vector pEASY T3 was used as a negative control. Precultures of *E. coli* CM2 transformants were grown in LBO broths to OD_600 nm_ of 1.0. Then, 1% of the above precultures continue to be grown in LB broths or LBO broths supplemented with 0.025 μg/ml norfloxacin. Cell growth was ended within 24 h for CM2 and 48 h for KNabc, followed by the evaluation of OD_600 nm_. Each data point stands for the mean ± SD of three independent cultures. **(D)** Na^+^/H^+^ antiport activity was determined using the everted membrane vesicles from *E. coli* KNabc expressing each variant of D223, with wild-type MdrP as a positive control and the empty vector pEASY T3 as a negative control. Each data point stands for the mean ± SD of three independent measurements. Intracellular Na^+^ contents of *E. coli* KNabc expressing D223E were measured in the presence of **(E)** 0.2 M NaCl and **(F)** 50 mM NaCl with or without the addition of norfloxacin, using wild-type MdrP as a positive control and the empty vector pEASY T3 as a negative control. *E. coli* KNabc transformants were grown in LBO broths to OD_600 nm_ of 1.0, and aliquots of cells were preincubated in the presence of 200 mM NaCl or 50 mM NaCl within 60 min. At that time, intracellular Na^+^ contents reached the steady level. Then, 2 μg/ml norfloxacin was added to test the effect of norfloxacin on the intracellular Na^+^ contents. The intracellular Na^+^ contents were determined using an atomic absorption spectrophotometer AA-6650 (Shimadzu, Kyoto, Japan). Each data point stands for the mean ± SD of three independent measurements.

To analyze the role of D223 in Na^+^ or/and H^+^ translocation, we analyzed the Na^+^- or H^+^-coupled norfloxacin efflux activity and Na^+^/H^+^ antiport activity of D223A, D223E, or D223N using the same assays to those for the variants of D244. In contrast, D223A reduced but retained, to greater extent, H^+^-coupled norfloxacin efflux activity (Figure 8A), whereas this variant almost lost Na^+^-coupled norfloxacin efflux activity (Figure 8B). The mimicking permanent protonation, D223N, completely or almost lost H^+^- and Na^+^-coupled norfloxacin efflux activity (Figures 8A,B). However, D223E almost lost H^+^-coupled norfloxacin efflux activity (Figure 8A) but restored the similar Na^+^-coupled norfloxacin efflux activity to that of wild-type MdrP (Figure 8B). This reveals that MdrP can be modified into a drug/Na^+^ antiporter with the weak drug/H^+^ antiport activity through the substitution of D223 by glutamic acid. This was confirmed by the result that D223E could not offer the resistance of *E. coli* CM2 to 0.025 μg/ml norfloxacin when LB both containing ∼0.17 M NaCl was replaced with LBO both without NaCl (Figure 8C). D223A, D223E, or D223N lost Na^+^/H^+^ antiport activity as compared with wild-type MdrP (Figure 8D). This suggests that D223 may be simultaneously involved in Na^+^ and H^+^ translocation. However, D223E mainly retained Na^+^-coupled norfloxacin efflux activity (Figure 8B), suggesting that substitution of D223 by glutamic acid may change the ionic coupling of norfloxacin efflux to a preference for Na^+^. Therefore, D223 may play a determining role in the participation in Na^+^ translocation other than H^+^ translocation.

To clarify this point, D223E was further selected to analyze the effect of norfloxacin treatment on intracellular Na^+^ contents of *E. coli* KNabc expressing this variant in the presence of 0.2 M NaCl, using wild-type MdrP as a positive control and the empty vector as a negative control. However, KNabc/D223E with or without norfloxacin treatment accumulated significantly higher intracellular Na^+^ content than those in KNabc/empty vector in the presence of 0.2 M NaCl (Figure 8E). In addition, there was the same positive linear correlationship between intracellular Na^+^ content and incubation time when KNabc/D223E was treated with or without norfloxacin (Figure 8E). Under the stress of 0.2 M NaCl, wild-type MdrP is the sole Na^+^/H^+^ antiporter that can maintain the balanced intracellular Na^+^ accumulation of *E. coli* KNabc. Therefore, KNabc/MdrP showed significantly lower intracellular Na^+^ content in the absence of norfloxacin than those in KNabc/empty vector with or without norfloxacin treatment (Figures 3C, 8E). However, because 2 μg/ml excessive norfloxacin was used in the intracellular Na^+^ accumulation assay, MdrP was very likely to have to mainly exhibit norfloxacin/Na^+^ antiport activity and masked the reduction in intracellular Na^+^ content induced by Na^+^/H^+^ antiport activity so that the extrusion of norfloxacin led to the influx of excessive Na^+^ into the cells of *E. coli* KNabc (Figures 3C, 8E). Notably, D223E retained the same norfloxacin/Na^+^ antiport activity with that of wild-type MdrP (Figure 8B), while this variant lost norfloxacin/H^+^ antiport activity (Figure 8A) and Na^+^/H^+^ antiport activity (Figure 8D). If D223E functioned only as a norfloxacin/Na^+^ antiporter, KNabc/D223E would at most show the same intracellular Na^+^ content with that of KNabc/MdrP in the presence of norfloxacin. The sole reasonable explanation is that D223E may lead to the leakage of excessive Na^+^ into the cells of *E. coli* KNabc in the presence of 0.2 M NaCl. Whether D223E can function as a norfloxacin/Na^+^ antiporter seems to be unable to be judged by analyzing the effect of norfloxacin treatment on intracellular Na^+^ content of KNabc/D223E in the presence of 0.2 M NaCl.

To confirm that D223E can function as a norfloxacin/Na^+^ antiporter, a significant difference in intracellular Na^+^ contents of KNabc/D223E with or without norfloxacin treatment needs to be detected. Therefore, 50 mM NaCl was selected to be used for incubating the tested cells of *E. coli* KNabc to avoid too high basal value. Since *E. coli* KNabc can grow in the presence of 50 mM NaCl via some low-activity Na^+^/H^+^ antiporters such as MdfA (Edgar and Bibi, 1997), MdtM (Holdsworth and Law, 2013), etc., it is reasonable that *E. coli* KNabc transformed with the empty vector can maintain lower intracellular Na^+^ content in the presence of 50 mM NaCl (Figure 8F) than the one in the presence of 0.2 M NaCl (Figure 8E). Wild-type MdrP can function not only as a norfloxacin/Na^+^ antiporter (Figure 3C) but also as a Na^+^/H^+^ antiporter (Figure 8D). Therefore, KNabc/MdrP with norfloxacin treatment accumulated significantly higher intracellular Na^+^ content than that without norfloxacin treatment in the presence of 50 mM NaCl (Figure 8F). In addition, it is reasonable that KNabc/MdrP accumulated significantly lower intracellular Na^+^ content than those in KNabc/empty vector under the same tested conditions (Figure 8F). KNabc/D223E without norfloxacin treatment accumulated the same intracellular Na^+^ content as those in KNabc/empty vector with or without norfloxacin treatment (Figure 8F), since D223E lost Na^+^/H^+^ antiport activity (Figure 8D). This also implies that low-activity Na^+^/H^+^ antiporters of *E. coli* KNabc can maintain intracellular Na^+^ content at the tolerable level in the presence of 50 mM NaCl, even if D223E without Na^+^/H^+^ antiport activity led to the leakage of extra Na^+^ into the cells of *E. coli* KNabc. Importantly, KNabc/D223E with norfloxacin treatment accumulated significantly higher intracellular Na^+^ content than that without norfloxacin treatment in the presence of 50 mM NaCl (Figure 8F). This indicates that D223E indeed functions as a norfloxacin/Na^+^ antiporter.

Based on the above results, we propose that D233 could mainly act as a key determinant in the Na^+^ translocation coupled to norfloxacin efflux, although this acidic residue may be involved in H^+^ translocation.

### Partial Effect of E188 and E341 in the Na^+^/H^+^ Antiport Activity

We also attempted to analyze the roles of E188 and E341 in the function due to their full conservation (Supplementary Figure S2) and important locations in the modeled structure of MdrP (Supplementary Figures S3A,B). However, substitution of E188 or E341 by alanine did not affect the resistance of MdrP to 0.025 μg/ml norfloxacin (Supplementary Figure S4A) but partially reduced the tolerance of MdrP to 0.2 M NaCl (Supplementary Figure S4B), suggesting that E188 and E341 may affect Na^+^/H^+^ antiport activity of MdrP, to some extent.

## Discussion

This study provides a strong evidence for the function of MdrP as a novel Na^+^-coupled MFS-MDR transporter. This transporter can extrude norfloxacin in a mode of drug/Na^+^ antiport and also may accomplish H^+^-coupled norfloxacin efflux independently of Na^+^. More importantly, we propose that MdrP employs D223 as a key determinant in the Na^+^ translocation coupled to norfloxacin efflux, although this acidic residue may be involved in H^+^ translocation. In contrast, D127 and D244 may be involved in H^+^ or/and Na^+^ translocation. These findings remind the possibility that MFS-MDR transporters may employ Na^+^ to resist against superfluous drug, besides H^+^. Owing to the ubiquity of MFS-MDR transporters, the presented findings also have guiding significance to figure out the solution to drug resistance accompanied with medical treatment by bacterial infections. Furthermore, our results suggest that R71 in motif A, but not D67, forms the polar contacts with D127 in TMH4 and R361 in TMH11, which is responsible for the stabilization of the outward-facing conformation of MdrP. This partially modifies the knowledge of the conformation-related residues in motif A of MFS transporters and implies the importance of a new positively charged residue, R361, for the stabilization of outward-facing conformation of MFS transporters.

A canonical structure of MFS transporters is known as MFS fold, which is formed by the N and C domains with both composed of six consecutive TMHs, respectively (Jiang et al., 2013; Yan, 2013a). In *E. coli* YajR, an uncharacterized MFS transporter, D73 and R77 in motif A can form a charge-relay system with D126 in TMH4 and also may presumably regulate the strength of the charge-helix dipole interaction between D73 (for N domain) and TMH11 (for C domain), which locks the domain interface on the cytoplasmic side and achieves the control of stable outward-facing conformation of MFS fold (Yan, 2013a). D68 in motif A and D128 in TMH4 have been established to be two critical protonation sites in a transmembrane H^+^ relay pathway (D235 > E327 > D142 > D68 > D128) of *L. lactis* LmrP (Masureel et al., 2014). Our results support the existence of a charge-relay system between R71 (R77 in YajR) and D127 (D126 in YajR) (Figures 4, 5). Based on the prediction of polar contacts between residues, we speculate that D67, R71, D127, and R361 may be responsible for the stabilization of outward-facing conformation of MdrP. However, mutation in D67 (D73 in YajR and D68 in LmrP, respectively) did not affect the norfloxacin resistance or NaCl tolerance of MdrP (Figure 4C), indicating that this residue is not involved in the stabilization of outward-facing conformation of MdrP. Instead, D127 may directly participate in the polar contact with R361 in TMH11 (Figures 4B,C, 5), which is different from the conformation-stabilizing mechanism of *E. coli* YajR through the charge-helix dipole interaction between D73 in motif A and N terminus of TMH11 (Jiang et al., 2013). Altogether, our results highlight the pivotal role of D127, an acidic residue located out of motif A, in the whole conformation-stabilizing mechanism of MdrP. This residue may not only form a charge-relay system with R71 but also interact with R361 in TMH11 and thus achieves the stabilization of the domain interface on the cytoplasmic side. This is supported by the fact that the above three residues are simultaneously required by both Na^+^- or H^+^-coupled norfloxacin efflux and Na^+^/H^+^ antiport of MdrP (Figures 4C, 5).

MdrP can exhibit H^+^-coupled norfloxacin efflux activity independently of Na^+^ and also Na^+^/H^+^ antiport activity independently of norfloxacin. The discovery of MdrP provides an excellent transporter model for the recognition of Na^+^- or/and H^+^-coupled key acidic residues through the effects of site-directed mutagenesis on Na^+^/H^+^ antiport activity. For example, if mutation leads to the loss of Na^+^/H^+^ antiport activity, the corresponding residue may be involved in Na^+^ and H^+^ translocation. D244 and E341 of MdrP correspond to D235 and E327 of *L. lactis* LmrP, respectively (Figure 6A). D235 and E327 have been shown to be the two key acidic residues in a transmembrane H^+^ relay pathway (D235 > E327 > D142 > D68 > D128) of *L. lactis* LmrP (Masureel et al., 2014). Our results suggest that D244 may simultaneously participate in H^+^ and Na^+^ translocation, which is responsible for not only Na^+^/H^+^ antiport but also H^+^ and Na^+^ coupling to norfloxacin efflux (Figures 6C, 7). However, E341 may be inessential for H^+^ and Na^+^ translocation, since substitution of E341 by alanine affected Na^+^/H^+^ antiport activity of MdrP, to some extent (Supplementary Figure S4). The structurally elucidated MFS transporters show that the central cavity enclosed by the N and C domains is exposed outwardly to the solvent in the outward-facing state (Yan, 2015). The membrane-buried proline residues of transporters can interrupt the hydrogen bonding network of transmembrane α helices and thus allow the carbonyl oxygen of the residue at the −4 position of proline to participate in polar interactions with substrates including solutes, cations, or protons (Deber and Therien, 2002). A H^+^-coupled MATE transporter from *Pyrococcus furiosus* (PfMATE) has been shown to adopt two distinct conformations, a bent conformation and a straight conformation, in terms of the structure of TMH1 (Tanaka et al., 2013). A H^+^- and Na^+^-coupled MATE transporter, *E. coli* ClbM, was also proposed using its X-ray structure via molecular dynamics simulations to share two similar conformations to those of PfMATE (Mousa et al., 2016; Mousa et al., 2017; Krah et al., 2020). This structural shift is proposed to satisfy the requirement for protonation of a conserved aspartate residue in TMH1 between H^+^-coupled MATE transporters (Lu et al., 2013a; Tanaka et al., 2013). A kink induced by Pro26 or Pro38 has also been established to contribute to the bent conformation of TMH1 of PfMATE and *E. coli* ClbM, respectively (Tanaka et al., 2013; Mousa et al., 2016, 2017). D244 is located at the −4 position of P248 in TMH7 of MdrP (Figure 6A) and faces the central cavity in the middle of the modeled structure (Figure 6B). Therefore, D244 may serve as one of the most important protonation or Na^+^-binding sites in the central cavity of MdrP, which is responsible for the capture of protons and Na^+^ from the periplasmic side. In addition, P248 may play an important role in the possible conformational change of TMH7 nearby D244.

In secondary transporters, the negatively charged side chains from Glu and Asp are involved in cation coordination or protonation (Shi, 2013). In addition, the unwound segments of discontinuous transmembrane helices allow the carbonyl oxygens to escape the intrahelical hydrogen bonds to participate in coordination with cations other than protons (Shi, 2013). D223 is located in the unwound segment between Helix α 6–7 and TMH7 (Figures 6A,B and Supplementary Figure S2), which implies that this acidic residue may be vital for the Na^+^ translocation of MdrP. D223A lost drug/Na^+^ antiport activity and D223E mainly exhibited drug/Na^+^ antiport activity (Figures 8B,C), revealing that the negative charge of side chain from D233 could play a determining role in Na^+^ translocation and the extended side chain even may change the ionic coupling of norfloxacin efflux to a preference for Na^+^. Furthermore, D223N mimicked the permanent protonation and therefore blocked H^+^ release from D223 (Figures 8A,B), supporting that this acidic residue is also involved in H^+^ translocation. This is consistent with the canonical antiport mechanism of secondary transporters wherein coupling ions compete with positively charged substrates for the shared binding sites (Hunte et al., 2005; Arkin et al., 2007; Adam et al., 2007; Chen et al., 2007). This explains why D223E abolished Na^+^/H^+^ antiport activity (Figure 8D) when it retained drug/Na^+^ antiport activity (Figures 8B,C). This is because glutamic acid at No. 223 residue position prefers to bind with Na^+^ rather than H^+^ and therefore disrupts the competitive binding balance between Na^+^ and H^+^. It is possible that the introduction of the longer side chain to D223 as the optimal binding site for protons may have been affected. Thus, substitution of D223 by glutamic acid may not only lead to the loss of Na^+^/H^+^ antiport activity of MdrP (Figure 8D) but also modify the function of MdrP from a Na^+^/H^+^ antiporter into a Na^+^ channel that only may passively diffuse Na^+^ down the thermodynamic gradient without the coupling to H^+^ (Figure 8E). Interestingly, D223A retained, to great extent, H^+^-coupled norfloxacin efflux activity (Figure 8A), suggesting that the carbonyl oxygen of main chain from D223 may be involved in H^+^ translocation. However, the carbonyl oxygen of main chains from Glu and Asp of secondary transporters is proposed to be always involved in coordination with cations other than protons (Shi, 2013). Therefore, it seems possible that MdrP may achieve H^+^ translocation even without the aid of D223. Altogether, our results strongly support that D233 could mainly act as a key determinant in Na^+^ translocation coupled to norfloxacin efflux, although this acidic residue is also involved in H^+^ translocation.

**FIGURE 9:**
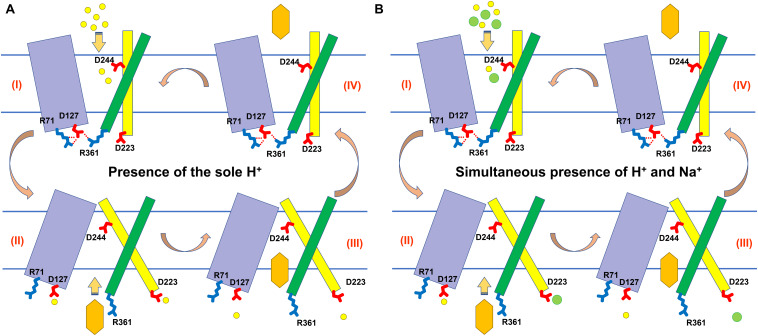
Proposed models of MdrP for Na^+^- or H^+^-coupled norfloxacin efflux. N domain composed of TMH1-6 is drawn as light blue rectangles, and TMH7 and TMH11 of C domain are drawn as yellow and green rectangles, respectively. Norfloxacin, H^+^, and Na^+^ are drawn as yellow sexangles, light yellow circles, and light green circles, respectively. **(A)** In the presence of the sole H^+^, D244 in outward-facing MdrP (state I) are simultaneously protonated; H^+^ is passed, respectively, in a relay mode of D244 > D127 and also in a relay mode of D244 > D223. The protonated D127 triggers the conformational switch of outward-facing MdrP to inward-facing state (II). Thus, inward-facing MdrP can accommodate norfloxacin in its “pocket” constituted by the N and C domains (III). The deprotonation of D127 results in the reversion of inward-facing MdrP to outward-facing state (IV). During this course, norfloxacin is simultaneously released from the pocket into the outside of cells. **(B)** However, in the simultaneous presence of H^+^ and Na^+^, the sole difference is that Na^+^ is solely passed from D244 to D223 and also released directly from D223.

It’s very interesting how H^+^ or Na^+^ is captured from the periplasmic side and then passed to the cytoplasmic side. D127 is exposed to the cytoplasm at the bottom of the central cavity of MdrP, whereas D223 is exposed to the cytoplasm at the C domain-membrane interface (Figures 4B, 6B and Supplementary Figures S3A,B). Based on the current results (Figures 4, 5), we speculate that the disruption or recovery of polar contacts not only between R71 and D127 but also between D127 and R361 may induce the conformational switch between inward- and outward-facing states (Supplementary Figures S3A,B). On account of this, D127 may act as the core conformation-stabilizing residue, similar to D126 in YajR (Jiang et al., 2013) and D128 in *L. lactis* LmrP (Masureel et al., 2014). Therefore, it is difficult to judge whether D127 is involved in the protonation, Na^+^ coordination or both, only based on the activity assays. Considering *L. lactis* LmrP being one of two phylogenetically closest homologs, we attempted to hypothesize that MdrP possess one similar ionic translocation pathway to that of LmrP (Masureel et al., 2014), which may be composed of D244, D223, D127, and possible other acidic residues. However, D223A retained the partial H^+^-coupled norfloxacin efflux activity (Figure 8A), supporting the possibility that MdrP may achieve H^+^ translocation even without the aid of D223. That is to say, H^+^ translocation from D224 to D127 may not require the relay of D223. Notably, D223E may lead to the leakage of excessive Na^+^ into the cells of *E. coli* KNabc (Figure 8E), implying that the extracellular Na^+^ may be directly released from D223 to the cytoplasm to support the drug/Na^+^ antiport. This also suggests that MdrP may possess two independent ionic traffic paths for H^+^ or/and Na^+^ translocation (Figure 6B): (i) H^+^ may be passed from D244 to D127 via the relay of other protonatable residue(s) in the central cavity; (ii) Na^+^ and partial H^+^ may be passed from D244 to D223 via the relay of other residue(s) in the C domain. *N. gonorrhoeae* NorM, an Na^+^-coupled MATE transporter, is proposed to translocate Na^+^ in the C domain accompanied with a local conformational change of TMH7 and TMH8 (Lu et al., 2013b). This enlightens us that MdrP may possess a similar Na^+^ translocation in the C domain to that of *N. gonorrhoeae* NorM, since MdrP also functions as a Na^+^-coupled MDR transporter. Thus, H^+^ may undergo a mainstream transmission from D244 to D127 through the central cavity of MdrP after it is captured by D244 (Figure 9), since the main conformational switch under the control of R71, D127, and R361 is the prerequisite for both Na^+^- or H^+^-coupled norfloxacin efflux and Na^+^/H^+^ antiport (Figures 4, 5). In addition, MdrP exhibited H^+^-coupled norfloxacin efflux activity independently of Na^+^, and the activity was significantly enhanced by Na^+^ (Figures 1D, 2D). Na^+^ and partial H^+^ may also be passed from D244 to D223 only in the C domain to induce a local intra-C domain conformational change, which may satisfy the requirement for Na^+^-coupled norfloxacin efflux and Na^+^/H^+^ antiport (Figure 9). This was also supported by the fact that D244 may also be involved in Na^+^ translocation (Figures 6C, 7) and D223 could mainly act as a key determinant in Na^+^ translocation (Figures 6C, 8).

The discovery of MdrP provides an excellent transporter model for Na^+^ and H^+^ coupling mechanisms of MFS transporters, even secondary transporters. It should be pointed out that the above suggestions are very interesting but based mainly on measurements in intact cells. In the future study, we plan to be assured of the conclusions using assays with a pure reconstituted protein. In addition, we plan to clarify whether MdrP possesses two independent ionic traffic paths for H^+^ or/and Na^+^ translocation. Furthermore, it is essential to search other key relay residues to confirm the detailed ionic traffic pathway for H^+^ or/and Na^+^ translocation. As a novel Na^+^-coupled MFS-MDR transporter, we also expect to overexpress and purify MdrP to discover its crystal structure and clarify its Na^+^ and H^+^ coupling molecular mechanism during drug efflux.

## Data Availability Statement

All datasets generated for this study are included in the article/Supplementary Material.

## Author Contributions

RZ and JJ contributed to the whole study design, analyzed the data, and revised the manuscript. RZ performed the gene subcloning, the assays for norfloxacin efflux accumulation, Na^+^/H^+^ antiport, and drafted the manuscript. RZ, QZ, and XZ performed the site-directed mutagenesis. RZ, QZ, SG, and YW measured the intracellular Na^+^ contents. RZ, HA-M, QZ, SG, YW, ZZ, LM, and TX contributed to the growth tests, preparation of everted membrane vesicles, and Western blot analysis. All the authors read and approved the final version of the manuscript.

## Conflict of Interest

The authors declare that the research was conducted in the absence of any commercial or financial relationships that could be construed as a potential conflict of interest.

## References

[B1] Abdel-Motaal H. Meng L. Zhang Z. Abdelazez A. H. Shao L. Xu T. (2018). An uncharacterized major facilitator superfamily transporter from *Planococcus maritimus* exhibits dual functions as a Na+(Li+, K+)/H+ antiporter and a multidrug efflux pump. *Front. Microbiol.* 9:1601. 10.3389/fmicb.2018.01601 30061877 PMC6055358

[B2] Adam Y. Tayer N. Rotem D. Schreiber G. Schuldiner S. (2007). The fast release of sticky protons: kinetics of substrate binding and proton release in a multidrug transporter. *Proc. Natl. Acad. Sci. U.S.A.* 104 17989–17994. 10.1073/pnas.0704425104 17984053 PMC2084284

[B3] Arkin I. T. Xu H. Jensen M. Arbely E. Bennett E. R. Bowers K. J. (2007). Mechanism of Na+/H+ antiporting. *Science* 317 799–803. 10.1126/science.1142824 17690293

[B4] Bay D. C. Rommens K. L. Turner R. J. (2008). Small multidrug resistance proteins: a multidrug transporter family that continues to grow. *Biochim. Biophys. Acta* 1778 1814–1838. 10.1016/j.bbamem.2007.08.015 17942072

[B5] Bolhuis H. Poelarends G. van Veen H. W. Poolman B. Driessen A. J. Konings W. N. (1995). The Lactococcal *lmrP* gene encodes a proton motive force-dependent drug transporter. *J. Biol. Chem.* 270 26092–26098. 10.1074/jbc.270.44.26092 7592810

[B6] Bradford M. M. (1976). A rapid and sensitive method for the quantitation of microgram quantities of protein utilizing the principle of protein-dye binding. *Anal. Biochem.* 72 248–254. 10.1016/0003-2697(76)90527-3 942051

[B7] Brown M. H. Paulsen I. T. Skurray R. A. (1999). The multidrug efflux protein NorM is a prototype of a new family of transporters. *Mol. Microbiol.* 31 394–395. 10.1046/j.1365-2958.1999.01162.x 9987140

[B8] Chen Y. Pornillos O. Lieu S. Ma C. Chen A. P. Chang G. (2007). X-ray structure of EmrE supports dual topology model. *Proc. Natl. Acad. Sci. U.S.A.* 104 18999–19004. 10.1073/pnas.0709387104 18024586 PMC2141897

[B9] Deber C. M. Therien A. G. (2002). Putting the β-breaks on the membrane protein misfoding. *Nat. Struct. Biol.* 9 318–319. 10.1038/nsb0502-318 11976722

[B10] Dong P. Wang L. Song N. Yang L. Chen J. Yan M. (2017). A UPF0118 family protein with uncharacterized function from the moderate halophile *Halobacillus andaensis* represents a novel class of Na+(Li+)/H+ antiporter. *Sci. Rep.* 7:45936. 10.1038/srep45936 28374790 PMC5379678

[B11] Edgar R. Bibi E. (1997). MdfA, an *Escherichia coli* multidrug resistance protein with an extraordinarily broad spectrum of drug recognition. *J. Bacteriol.* 179 2274–2280. 10.1128/jb.179.7.2274-2280.1997 9079913 PMC178964

[B12] Ethayathulla A. S. Yousef M. S. Amin A. Leblanc G. Kaback H. R. Guan L. (2014). Structure-based mechanism for Na+/melibiose symport by MelB. *Nat. Commun.* 5:3009. 10.1038/ncomms4009 24389923 PMC4026327

[B13] Fluman N. Bibi E. (2009). Bacterial multidrug transport through the lens of the major facilitator superfamily. *Biochim. Biophys. Acta* 1794 738–747. 10.1016/j.bbapap.2008.11.020 19103310

[B14] Griffith J. K. Baker M. E. Rouch D. A. Page M. G. P. Skurray R. A. Paulsen I. T. (1992). Membrane transport proteins: implications of sequence comparisons. *Curr. Opin. Cell Biol.* 4 684–695. 10.1016/0955-0674(92)90090-Y 1419050

[B15] Hassan K. A. Elbourne L. D. H. Li L. Gamage K. A. H. Liu Q. Jackson S. M. (2015). An ace up their sleeve: a transcriptomic approach exposes the AceI eflux protein of *Acinetobacter baumannii* and reveals the drug eflux potential hidden in many microbial pathogens. *Front. Microbiol.* 6:333 10.3389/fmicb.2015.00333 PMC4406071 25954261

[B16] He X. Szewczyk P. Karyakin A. Evin M. Hong W. Zhang Q. (2010). Structure of a cation-bound multidrug and toxic compound extrusion transporter. *Nature* 467 991–994. 10.1038/nature09408 20861838 PMC3152480

[B17] Higgins C. F. (2007). Multiple molecular mechanisms for multidrug resistance transporters. *Nature* 446 749–757. 10.1038/nature05630 17429392

[B18] Holdsworth S. R. Law C. J. (2013). Multidrug resistance protein MdtM adds to the repertoire of antiporters involved in alkaline pH homeostasis in *Escherichia coli*. *BMC Microbiol.* 13:113. 10.1186/1471-2180-13-113 23701827 PMC3668916

[B19] Hunte C. Screpanti E. Venturi M. Rimon A. Padan E. Michel H. (2005). Structure of a Na+/H+ antiporter and insights into mechanism of action and regulation by pH. *Nature* 435 1197–1202. 10.1038/nature03692 15988517

[B20] Jiang D. Zhao Y. Wang X. Fan J. Heng J. Liu X. (2013). Structure of the YajR transporter suggests a transport mechanism based on the conserved motif A. *Proc. Nat. Acad. Sci. U.S.A.* 110 14664–14669. 10.1073/pnas.1308127110 23950222 PMC3767500

[B21] Jin Y. Nair A. van Veen H. W. (2014). Multidrug transport protein NorM from *Vibrio cholerae* simultaneously couples to sodium- and proton-motive force. *J. Biol. Chem.* 289 14624–14632. 10.1074/jbc.m113.546770 24711447 PMC4031518

[B22] Krah A. Huber R. G. Zachariae U. Bond J. (2020). On the ion coupling mechanism of the MATE transporter ClbM. *Biochim. Biophys. Acta* 1862:183137. 10.1016/j.bbamem.2019.183137 31786188

[B23] Krah A. Zachariae U. (2017). Insights into the ion-coupling mechanism in the MATE transporter NorM-VC. *Phys. Biol.* 14:045009. 10.1088/1478-3975/aa5ee7 28169223

[B24] Kuroda T. Tsuchiya T. (2009). Multidrug efflux transporters in the MATE family. *Biochim. Biophys. Acta* 1794 763–768. 10.1016/j.bbapap.2008.11.012 19100867

[B25] Long F. Rouquette-Loughlin C. Shafer W. M. Yu E. W. (2008). Functional cloning and characterization of the multidrug efflux pumps NorM from *Neisseria gonorrhoeae* and YdhE from *Escherichia coli*. *Antimicrob. Agents Chemother.* 52 3052–3060. 10.1128/AAC.00475-08 18591276 PMC2533508

[B26] Lu M. Radchenko M. Symersky J. Nie R. Guo Y. (2013a). Structural insights into H+-coupled multidrug extrusion by a MATE transporter. *Nat. Struc. Mol. Biol.* 20 1310–1317. 10.1038/nsmb.2687 PMC3825517 24141706

[B27] Lu M. Symersky J. Radchenko M. Koide A. Guo Y. Nie R. (2013b). Structures of a Na+-coupled, substrate-bound MATE multidrug transporter. *Proc. Natl. Acad. Sci. U.S.A.* 110 2099–2104. 10.1073/pnas.1219901110 23341609 PMC3568332

[B28] Lubelski J. Konings W. N. Driessen A. J. M. (2007). Distribution and physiology of ABC-type transporters contributing to multidrug resistance in bacteria. *Microbiol. Mol. Biol. Rev.* 71 463–476. 10.1128/mmbr.00001-07 17804667 PMC2168643

[B29] Masureel M. Martens C. Stein R. A. Mishra S. Ruysschaert J. M. Mchaourab H. S. (2014). Protonation drives the conformational switch in the multidrug transporter LmrP. *Nat. Chem. Biol.* 10 149–155. 10.1038/nchembio.1408 24316739 PMC4749020

[B30] Meng L. Meng F. Zhang R. Zhang Z. Dong P. Sun K. (2017). Characterization of a novel two-component Na+(Li+. K+)/H+ antiporter from *Halomonas zhaodongensis*. *Sci. Rep.* 7:4221. 10.1038/s41598-017-04236-0 28652569 PMC5484666

[B31] Mine T. Morita Y. Kataoka A. Mizushima T. Tsuchiya T. (1998). Evidence for chloramphenicol/H+ antiport in Cmr (MdfA) system of *Escherichia coli* and properties of the antiporter. *J. Biochem.* 124 187–193. 10.1093/oxfordjournals.jbchem.a022078 9644262

[B32] Minhas G. S. Bawdon D. Herman R. Rudden M. Stone A. P. James A. G. (2018). Structural basis of malodour precursor transport in the human axilla. *eLife* 7:e34995. 10.7554/elife.34995 29966586 PMC6059767

[B33] Morita Y. Kataoka A. Shiota S. Mizushima T. Tsuchiya T. (2000). NorM of *Vibrio parahaemolyticus* is an Na+-driven multidrug efflux pump. *J. Bacteriol.* 182 6694–6697. 10.1128/JB.182.23.6694-6697.2000 11073914 PMC111412

[B34] Morita Y. Kodama K. Shiota S. Mine T. Kataoka A. Mizushima T. (1998). NorM, putative multidrug efflux protein, of *Vibrio parahaemolyticus* and its homolog in *Escherichia coli*. *Antimicrob. Agents Chemother.* 42 1778–1782. 9661020 10.1128/aac.42.7.1778 PMC105682

[B35] Mousa J. J. Newsome R. C. Yang Y. Jobin C. Bruner S. D. (2017). ClbM is a versatile, cation-promiscuous MATE multi-drug transporter found in the colibactin biosynthetic gene cluster. *Biochem. Biophys. Res. Comm.* 482 1233–1239. 10.1016/j.bbrc.2016.12.018 27939886

[B36] Mousa J. J. Yang Y. Tomkovich S. Shima A. Newsome R. C. Tripathi P. (2016). MATE transport of the *E. coli-*derived genotoxin colibactin. *Nat. Microbiol.* 1:15009. 10.1038/NMICROBIOL.2015.9 27571755 PMC5704960

[B37] Nishima W. Mizukami W. Tanaka Y. Ishitani R. Nureki O. Sugita Y. (2016). Mechanisms for two-step proton transfer reactions in the outward-facing form of MATE transporter. *Biophys. J.* 110 1346–1354. 10.1016/j.bpj.2016.01.027 27028644 PMC4816688

[B38] Nozaki K. Inaba K. Kuroda T. Tsuda M. Tsuchiya T. (1996). Cloning and sequencing of the gene for Na+/H+ antiporter of *Vibrio parahaemolyticus*. *Biochem. Biophys. Res. Commun.* 222 774–779. 10.1006/bbrc.1996.0820 8651921

[B39] Paulsen I. T. Brown M. H. Skurray R. A. (1996). Proton-dependent multidrug efflux systems. *Microbiol. Rev.* 60 575–608. 8987357 10.1128/mr.60.4.575-608.1996 PMC239457

[B40] Reddy V. S. Shlykov M. A. Castillo R. Sun E. I. Saier M. H. (2012). The Major Facilitator Superfamily (MFS) revisited. *FEBS J.* 279 2022–2035. 10.1111/j.1742-4658.2012.08588.x 22458847 PMC3425384

[B41] Saier M. H. Reddy V. S. Tsu B. V. Ahmed M. S. Li C. Moreno-Hagelsieb G. (2016). The transporter classification database (TCDB): recent advances. *Nucleic Acids Res.* 44 D372–D379. 10.1093/nar/gkv1103 26546518 PMC4702804

[B42] Shao L. Abdel-Motaal H. Chen J. Chen H. Xu T. Meng L. (2018). Characterization of a functionally unknown arginine-aspartate-aspartate family protein from *Halobacillus andaensis* and functional analysis of its conserved arginine/aspartate residues. *Front. Microbiol.* 9:807. 10.3389/fmicb.2018.00807 29922240 PMC5996927

[B43] Shi Y. (2013). Common folds and transport mechanisms of secondary active transporters. *Annu. Rev. Biophys.* 42 51–72. 10.1146/annurev-biophys-083012-130429 23654302

[B44] Tanaka Y. Hipolito C. J. Maturana A. D. Ito K. Kuroda T. Higuchi T. (2013). Structural basis for the drug extrusionmechanismby a MATE multidrug transporter. *Nature* 496 247–251. 10.1038/nature12014 23535598

[B45] Tseng T. T. Gratwick K. S. Kollman J. Park D. Nies D. H. Goffeau A. (1999). The RND permease puperfamily: an ancient, ubiquitous and diverse family that includes human disease and development proteins. *Mol. Microbiol. Biotechnol.* 1 107–125. 10941792

[B46] Wang Y. Song N. Yang L. Abdel-motaal H. Zhang R. Zhang Z. (2017). A novel NhaD-type Na+/H+ antiporter from the moderate halophile and alkaliphile *Halomonas alkaliphila*. *Can. J. Microbiol.* 63 596–607. 10.1139/cjm-2017-0104 28329448

[B47] Wong K. Ma J. Rothnie A. Biggin P. C. Kerr I. D. (2014). Towards understanding promiscuity in multidrug efflux pumps. *Trends Biochem. Sci.* 39 8–16. 10.1016/j.tibs.2013.11.002 24316304

[B48] Xu T. Chen H. Li J. Hong S. Shao L. Zheng X. (2019). Implications for cation selectivity and evolution by a novel cation diffusion facilitator family member from the moderate halophile *Planococcus dechangensis*. *Front. Microbiol.* 10:607. 10.3389/fmicb.2019.00607 30967858 PMC6440370

[B49] Yan N. (2013a). Structural advances for the major facilitator superfamily (MFS) transporters. *Trends Biochem. Sci.* 38 151–159. 10.1016/j.tibs.2013.01.003 23403214

[B50] Yan N. (2013b). Structural investigation of the proton-coupled secondary transporters. *Curr. Opin. Struct. Biol.* 23 483–491. 10.1016/j.sbi.2013.04.011 23806360

[B51] Yan N. (2015). Structural biology of the major facilitator superfamily transporters. *Annu. Rev. Biophys.* 44 257–283. 10.1146/annurev-biophys-060414-033901 26098515

