# Supplementary material for: A Novel MFS-MDR Transporter, MdrP, Employs D223 as a Key Determinant in the Na+ Translocation Coupled to Norfloxacin Efflux
Source: Front Microbiol. 2020 May 29;11:955. doi: 10.3389/fmicb.2020.00955 (PMC7272687; doi:10.3389/fmicb.2020.00955)
Supplement: Supplementary file 1 [file Data_Sheet_1.pdf]

Supplementary Material

A novel MFS-MDR transporter, MdrP, employs D223 as a key determinant in the Na^+ translocation coupled to norfloxacin efflux

Rui Zhang, Heba Abdel-Motaal, Qiao Zou, Sijia Guo, Xiutao Zheng, Yuting Wang, Zhenglai Zhang, Lin Meng, Tong Xu, Juquan Jiang*[*]

Department of Microbiology and Biotechnology, College of Biological Sciences, Northeast Agricultural University,Harbin, China

*** Correspondence:**
Juquan Jiang
jjqdainty@163.com

Supplemental Table 1. Primers used in this study.

Primers	Primer sequence (5' to 3')	The source
MdrP-F	TCTAGAGTGTCTTTGGAAACGGGCAG	This study
MdrP-R	GGTACCTTAATGATGATGATGATGATGCTTGTCGTCGTCGTCTAT GTTCGCAACCGTTCTTTATC (6×His tag, underlined)	This study
YdhE-F	TTGTCACCCACCAGCGCGGA	This study
YdhE-R	ATAATATGACCTGACATTAA	This study
NhaD-F	TATACCTACAAAGCAACGGA	This study
NhaD-R	AAAGCGCTTACCGCTGGATG	This study
D67A-F	GGCTATTTTGCTGCACGGTTCGGGC (Mutagenic bases, underlined)	This study
D67A-R	TGCAGCAAAATAGCCGCCAAATAAA (Mutagenic bases, underlined)	This study
R71A-F	GACCGGTTCGGGGCCAAGCGGATGC (Mutagenic bases, underlined)	This study
R71A-R	GGCCCCGAACCGGTCAGCAAAATAG (Mutagenic bases, underlined)	This study
R71K-F	GACCGGTTCGGGAAGAAGCGGATGC(Mutagenic bases, underlined)	This study
R71K-R	CTTCCCGAACCGGTCAGCAAAATAG (Mutagenic bases, underlined)	This study
D127A-F	GCAATGATTGCCGCTGTCATTCCTG (Mutagenic bases, underlined)	This study
D127A-R	AGCGGCAATCATTGCTTGGCTCGCT (Mutagenic bases, underlined)	This study
D127E-F	GCAATGATTGCCGAGGTCATTCCTG (Mutagenic bases, underlined)	This study
D127E-R	CTCGGCAATCATTGCTTGGCTCGCT (Mutagenic bases, underlined)	This study
D127N-F	GCAATGATTGCCAATGTCATTCCTG (Mutagenic bases, underlined)	This study
D127N-R	ATTGGCAATCATTGCTTGGCTCGCT (Mutagenic bases, underlined)	This study
E188A-F	TTTTATACAGAGGCGACTTTATCGG (Mutagenic bases, underlined)	This study
E188A-R	CGCCTCTGTATAAAAGCGTAATAAC (Mutagenic bases, underlined)	This study
D223A-F	ATCATTTTAAAAGCCCGTGTCTTTT (Mutagenic bases, underlined)	This study
D223A-R	GGCTTTTAAAATGATGCCGTAATCT (Mutagenic bases, underlined)	This study
D223E-F	ATCATTTTAAAAGAGCGTGTCTTTT (Mutagenic bases, underlined)	This study
D223E-R	CTCTTTTAAAATGATGCCGTAATCT (Mutagenic bases, underlined)	This study
D223N-F	ATCATTTTAAAAAATCGTGTCTTTT (Mutagenic bases, underlined)	This study
D223N-R	ATTTTTTAAAATGATGCCGTAATCT (Mutagenic bases, underlined)	This study
D244A-F	TTCATGCAATTGGCGCTCGTTATTC (Mutagenic bases, underlined)	This study
D244A-R	CGCCAATTGCATGAAAGTCTGCGCC (Mutagenic bases, underlined)	This study
D244E-F	TTCATGCAATTGGAACTCGTTATTC (Mutagenic bases, underlined)	This study

D244E-R	TTCCAATTGCATGAAAGTCTGCGCC (Mutagenic bases, underlined)	This study
D244N-F	TTCATGCAATTGAACCTCGTTATTC (Mutagenic bases, underlined)	This study
D244N-R	GTTCAATTGCATGAAAGTCTGCGCC (Mutagenic bases, underlined)	This study
E341A-F	TTCACATTTGCCGCATTGATGGTAG (Mutagenic bases, underlined)	This study
E341A-R	TGCGGCAAATGTGAAAACTGCCATC (Mutagenic bases, underlined)	This study
R361A-F	CCGGAGTCCATGGCCGGACAGTATT (Mutagenic bases, underlined)	This study
R361A-R	GGCCATGGACTCCGGCGCCAGTTTA (Mutagenic bases, underlined)	This study
R361K-F	CCGGAGTCCATGAAAGGACAGTATT (Mutagenic bases, underlined)	This study
R361K-R	TTTCATGGACTCCGGCGCCAGTTTA (Mutagenic bases, underlined)	This study

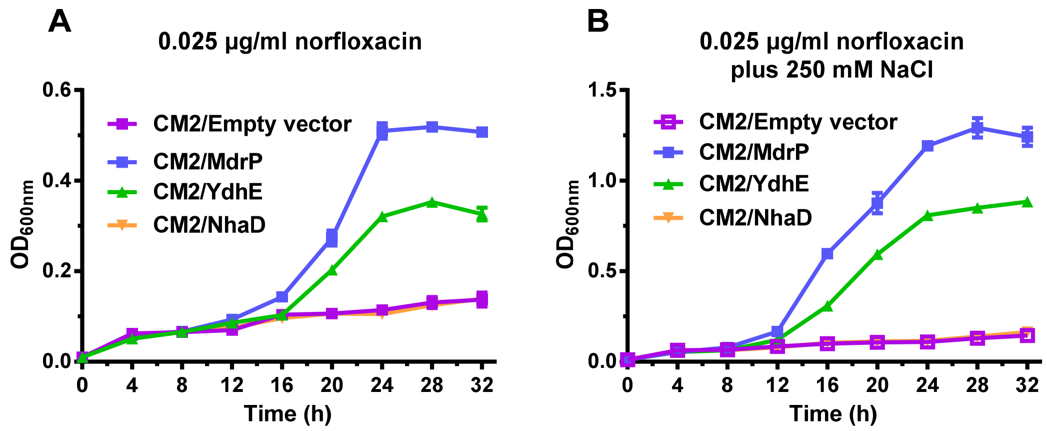

Supplemental Figure 1. Growth curves of *E. coli* CM2 transformants in the LBO media plus 0.025 μg/ml norfloxacin without or with the addition of NaCl.

Pre-cultures of *E. coli* CM2 transformants were grown in LBO broths to $OD_{600\ nm}$ of 1.0, and then 1% of pre-cultures were innoculated in the fresh LBO broths plus 0.025 μg/ml norfloxacin without the addition of NaCl (A) or with the addition of 250 mM NaCl (B). Growth curves were plotted by evaluating $OD_{600\ nm}$ of *E. coli* CM2 transformants in triplicate within 32 h. Blue filled square stands for CM2/MdrP; green filled upward triangle stands for CM2/YdhE as the positive control of a H^+-coupled norfloxacin efflux transporter; brown filled downward triangle stands for CM2/NhaD as the positive control of a Na^+/H^+ antiporter; purple open square stands for CM2/Empty vector as a negative control.

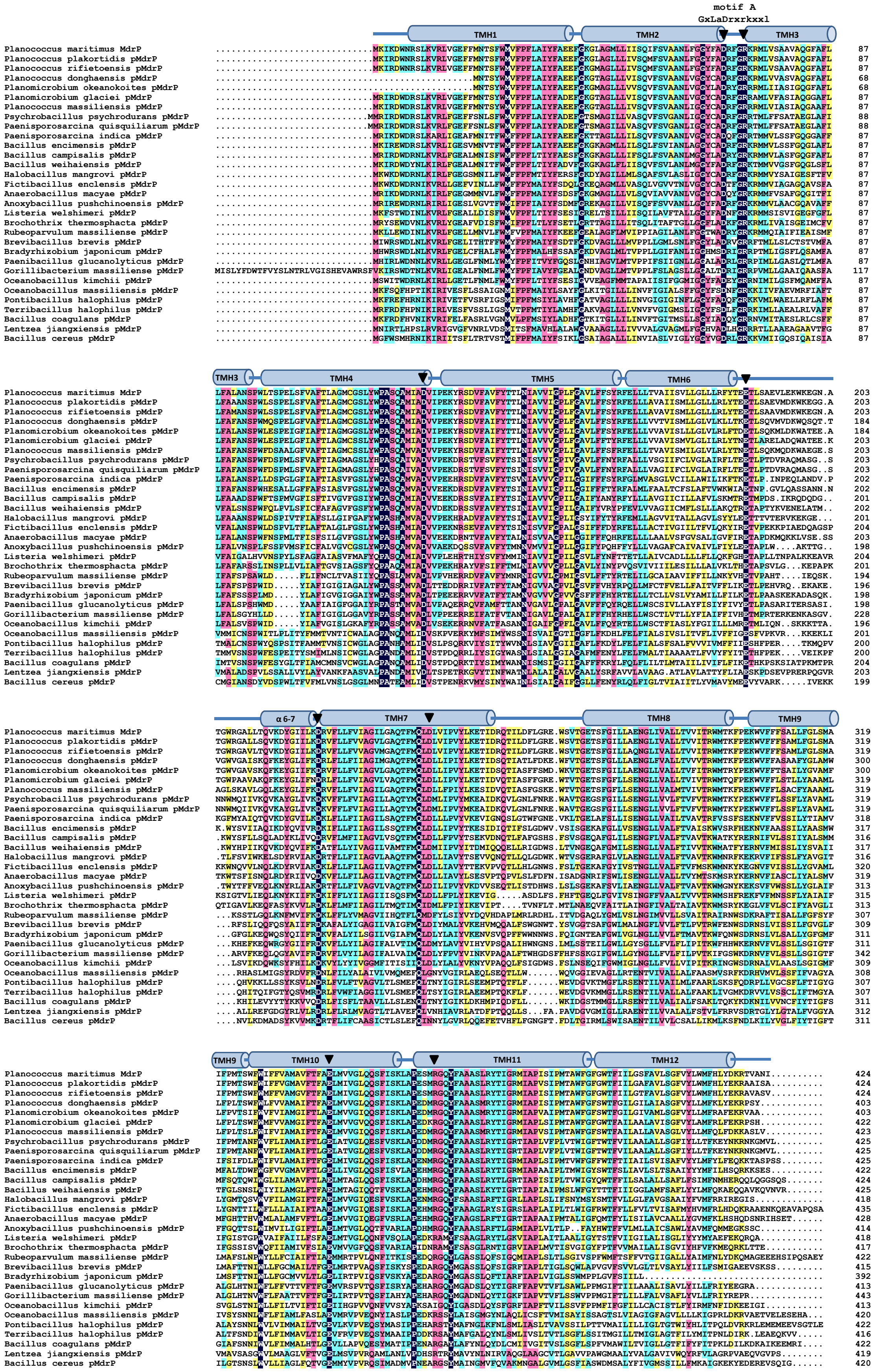
motif A
GxLaDrxrkxxl
TMH1
TMH2
TMH3
TMH4
TMH5
TMH6
α 6-7
TMH7
TMH8
TMH9
TMH10
TMH11
TMH12
Planococcus maritimus MdrP
Planococcus plakortidis pMdrP
Planococcus rifietoensis pMdrP
Planococcus donghaensis pMdrP
Planomicrobium okeanokoites pMdrP
Planomicrobium glaciei pMdrP
Planococcus massiliensis pMdrP
Psychrobacillus psychrodurans pMdrP
Paenisporosarcina quisquiliarum pMdrP
Paenisporosarcina indica pMdrP
Bacillus encimensis pMdrP
Bacillus campisalis pMdrP
Bacillus weihaiensis pMdrP
Halobacillus mangrovi pMdrP
Fictibacillus enclensis pMdrP
Anaerobacillus macyae pMdrP
Anoxybacillus pushchinoensis pMdrP
Listeria welshimeri pMdrP
Brochothrix thermosphacta pMdrP
Rubeoparvulum massiliense pMdrP
Brevibacillus brevis pMdrP
Bradyrhizobium japonicum pMdrP
Paenibacillus glucanolyticus pMdrP
Gorillibacterium massiliense pMdrP
Oceanobacillus kimchii pMdrP
Oceanobacillus massiliensis pMdrP
Pontibacillus halophilus pMdrP
Terribacillus halophilus pMdrP
Bacillus coagulans pMdrP
Lentzea jiangxiensis pMdrP
Bacillus cereus pMdrP

Supplemental Figure 2.Alignment of MdrP with 30 representatives of its homologs.

For the analysis of residue conservation, MdrP was aligned with 30 representatives of its homologs clustered within our recently reported phylogenetic tree (Abdel-Motaal et al., 2018). Shading homology corresponds to 100 % (black), ≥ 75 % (pink), ≥ 50 % (cyan) and ≥ 33 % (yellow) amino acid identity, respectively. The twelve putative transmembrane helices (TMHs) are marked with light blue cylinders above the alignment. The consensus sequence of the motif A was highlighted above the alignment between TMH2 and TMH3. Conserved amino acid residues (downward triangle) were selected for functional analysis via site-directed mutagenesis.

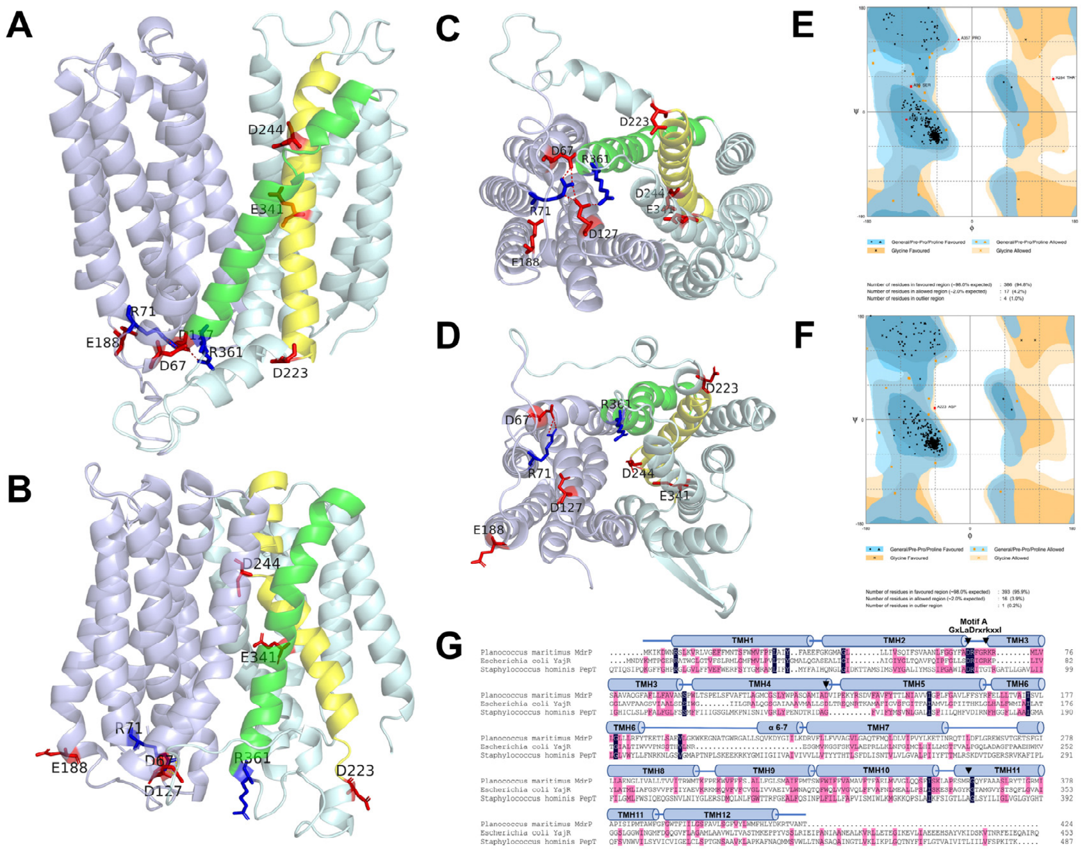

Supplemental Figure 3. Modeled structures of MdrP based on the templates of 3D structures of *E. coli* YajR and *Staphylococcus hominis* PepT.

The modeled structures of MdrP were predicted by using *E. coli* YajR 3D structure and *S. hominis* PepT 3D structureas the templates of outward-facingconformation and inward-facing one through the submission of its deduced amino acid sequence to the Phyre2 website http://www.sbg.bio.ic.ac.uk/~phyre2/html/page.cgi?id=index. TMH7-8 of PepT was considered to be a long loop and α helix 6-7 between TMH6 and TMH7 of MdrP when a modeled structure was constructed using PepT as a template.Predicted outward-facingand inward-facing conformation stereo views oriented parallel to the membrane (A&B)and from the periplasm (C&D) oriented vertical to the membrane were shown, respectively. Conserved residues between MdrP and 30 representatives of its homologs (Abdel-Motaal et al., 2018) were highlighted in color and labelled with their residue names, TMH7 and TMH11 were specially colored in yellow and green, respectively.The combinations of the ø and ψ angles of residues in favored, allowed and outlier regions of Ramachandran plot of outward-facingand inward-facing conformation (E&F) were qualified to test the reliability of structural analysis. Also, alignment of MdrP with *E. coli* YajR and *S. hominis* PepT (G) was carried out to show their identity and coverage, and four potential conformation-related residues were highlighted with downward triangles above the alignment.

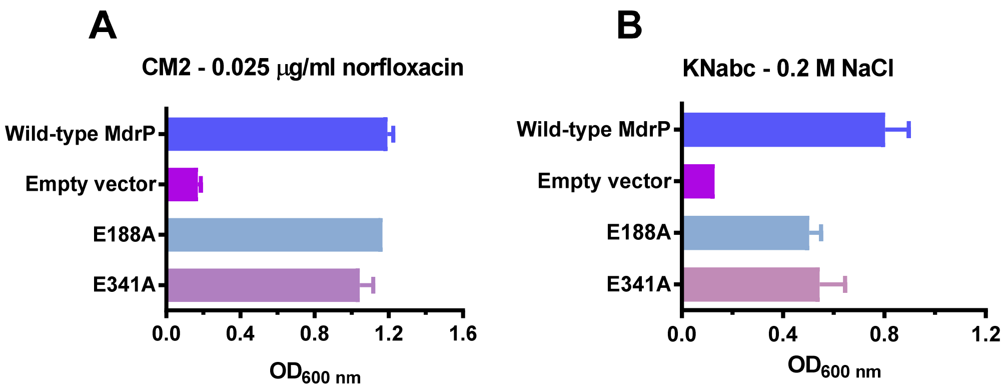

Supplemental Figure 4. Growth tests for norfloxacin or NaCl resistance of *E. coli* CM2 or KNabc expressing variants E188A and E341A.

The effect of each variant on norfloxacin resistance was tested by its complementation with *E. coli* CM2 in LB broths containing 0.025 µg/ml norfloxacin (A) or with *E. coli* KNabcinLBK broths containing 0.2 M NaCl (B). Cell growth was ended within 24 h for CM2 or 48 h for KNabc, followed by the evaluation of $OD_{600\ nm}$. Each data point stands for the mean ± SD of three independent cultures.
